# Liposome–trimethyl chitosan nanoparticles codeliver insulin and siVEGF to treat corneal alkali burns by inhibiting ferroptosis

**DOI:** 10.1002/btm2.10499

**Published:** 2023-02-09

**Authors:** Xiaojing Xiong, Huiting Jiang, Yukun Liao, Yangrui Du, Yu Zhang, Zhigang Wang, Minming Zheng, Zhiyu Du

**Affiliations:** ^1^ Department of Ophthalmology Second Affiliated Hospital of Chongqing Medical University Chongqing China; ^2^ Chongqing Key Laboratory of Ultrasound Molecular Imaging Second Affiliated Hospital of Chongqing Medical University Chongqing China; ^3^ State Key Laboratory of Ultrasound in Medicine and Engineering Second Affiliated Hospital of Chongqing Medical University Chongqing China

**Keywords:** corneal alkali burn, ferroptosis inhibition, insulin, nanomedicine, siVEGF

## Abstract

Alkali burns are potentially blinding corneal injuries. Due to the lack of available effective therapies, the prognosis is poor. Thus, effective treatment methods for corneal alkali burns are urgently needed. Codelivery nanoparticles (NPs) with characteristics such as high bioavailability and few side effects have been considered effective therapeutic agents for ocular diseases. In this study, we designed a new combination therapy using liposomes and trimethyl chitosan (TMC) for the codelivery of insulin (INS) and vascular endothelial growth factor small interfering RNA (siVEGF) to treat alkali‐burned corneas. We describe the preparation and characterization of siVEGF‐TMC‐INS‐liposome (siVEGF‐TIL), drug release characteristics, intraocular tracing, pharmacodynamics, and biosafety. We found that siVEGF‐TIL could inhibit oxidative stress, inflammation, and the expression of VEGF in vitro and effectively maintained corneal transparency, accelerated epithelialization, and inhibited corneal neovascularization (CNV) in vivo. Morever, we found that the therapeutic mechanism of siVEGF‐TIL is possibly relevant to the inhibition of the ferroptosis signaling pathway by metabolomic analysis. In general, siVEGF‐TIL NPs could be a safe and effective therapy for corneal alkali burn.

## 1 INTRODUCTION

Corneal alkali burns are one of the most common emergencies in ophthalmology, accounting for 11.5%–22.1% of all ocular traumas.^1^ As a result of corneal alkali injury, the ocular surface and anterior eye segment are extensively damaged, causing permanent vision impairment or even complete blindness.^2^ It has been reported that corneal oxidative stress occurs immediately after alkali damage, which precedes the corneal inflammatory response.^3^ During alkali burn‐induced injury, excessive oxidative stress in the cornea, oxidative changes occur in cellular macromolecules, and lipid peroxidation occurs in the membrane,^4^ leading to an antioxidant/pro‐oxidant imbalance in corneal tissues. On the other hand, the activity of antioxidant enzymes is decreased, while the expression and activity of catalytic enzymes running at physiological levels or even increases, leading to an increase in reactive oxygen species (ROS) production and a decrease in ROS decomposition.^5^ These factors can cause a high level of oxidative stress, eventually resulting in excessive intracorneal inflammation, scarring, and corneal neovascularization (CNV).^6^ Similar to oxidative stress, CNV plays a critical role in the pathophysiology of corneal alkali burns. CNV increases vascular permeability, which exacerbates inflammation, chronic edema, lipid exudation, and corneal scarring, potentially resulting in permanent vision loss.^7^

Currently, topical corticosteroids and nonsteroidal anti‐inflammatory drugs (NSAIDs) remain the top priorities. However, these treatments can delay wound healing, and long‐term use of corticosteroids can lead to increased intraocular pressure (IOP), cataracts, and an increased risk of infection.^8^ Even though various other treatment options have been available in the clinic, such as amniotic membrane transplantation, their effectiveness has not been optimal in the past two decades.^9^ Consequently, it is urgent to explore a more efficient and safe treatment for severe corneal alkali burns.

Insulin (INS), a hypoglycemic hormone, is mainly used in the clinical treatment of diabetes. However, a growing body of research has identified INS as a potential antioxidant. Ramalingam et al.^10^ demonstrated that INS pretreatment of cells could inhibit the cytotoxicity induced by H_2_O_2_, inhibiting apoptosis and increasing the PI3K/Akt survival pathway. Research by Rajasekar et al.^11^ proved INS could alleviate memory impairment in rats by reducing oxidative nitrogen stress. In fact, over the years, many studies have shown that INS can treat corneal injuries. Yang et al.^12^ identified that INS promoted corneal nerve repair and wound healing in mice with type 1 diabetes. Cruz‐Cazarim et al.^13^ showed that INS could treat dry eye syndrome and corneal injuries. Morever, a recent clinical study suggested that topical INS was an effective way to safely promote the healing of persistent epithelial defects in patients who were unresponsive to standard treatment.^14^ However, the use of INS in the treatment of corneal alkali burns has been rarely reported. We hypothesize that the local application of INS may be a promising strategy for the treatment of corneal alkali burns due to its antioxidant capacity.

In addition to inhibiting oxidative stress, the treatment of CNV is also essential for corneal alkali burns. CNV can be effectively treated by inhibiting vascular endothelial growth factor (VEGF) and its receptors, which modulate angiogenesis.^15^ Anti‐VEGF antibodies are therefore used to treat CNV, either through topical or subconjunctival applications.^16^ Unfortunately, anti‐VEGF antibodies are generally limited due to their poor efficacy, side effects, and drug resistance.^7^ RNA interference is a powerful approach to knocking down target genes.^17^ VEGF small interfering RNA (siVEGF) reduces VEGF expression and CNV.^18^, ^19^, ^20^, ^21^ Accordingly, it is reasonable to speculate that siVEGF could enhance the therapeutic effects of INS on corneal alkali injury, and codelivery of INS and siVEGF may provide a new combination therapy for corneal alkali injury.

It is well‐established that corneal physiological and physical barriers impair drug and siRNA penetration. To improve bioavailability, nanopharmaceuticals have been extensively developed to deliver siRNA or ocular drugs to treat ocular diseases.^22^, ^23^, ^24^ A wealth of studies have suggested that INS‐loaded liposomes could increase the bioavailability of INS.^25^, ^26^ However, the poor stability of liposomes leads to the rapid release of the encapsulated drugs, which impairs the therapeutic effects of drugs. Chitosan (CS) is a deacetylated pyran polysaccharide isolated from chitin that is biocompatible, nontoxic, and biodegradable and has been widely used to prepare nanocarriers such as micelles and nanoparticles (NPs). CS can also be used as a coating for liposomes to improve their stability in vitro and in vivo.^27^ Furthermore, CS can form NPs and be loaded with negatively charged nucleic acids and have been considered promising carriers for gene delivery.^28^ Morever, this encapsulation protects nucleic acids from host nucleases.^29^ As a quaternary CS derivative, trimethyl chitosan (TMC) also possesses these properties, and it is preferred due to its high water solubility, ionic stability, and cationic density.^30^

In this study, we developed a novel eye drop formulation based on liposomes and TMC to encapsulate and deliver INS and siVEGF. We expect these NPs to enhance the treatment efficacy of corneal alkali burns through the cooperative effects of INS and siVEGF, as well as their enduring effects and high bioavailability. We also explored the potential mechanism of the siVEGF‐TMC‐INS‐liposome (siVEGF‐TIL) NPs in treating corneal alkali burn.

## 2 MATERIALS AND METHODS

### 2.1 Materials and reagents

Human recombinant INS, perfluorooctyl bromide (PFOB), and 1,1′‐dioctadecyl‐3,3,3′,3′‐tetramethylindo‐tricarbocyanine iodide (DiR) were bought from Sigma‐Aldrich. 1,2‐Stearoyl‐sn‐glycerol‐3‐phosphoethanolamine‐N‐[methoxy (polyethylene glycol)‐2000] (DSPE‐mPEG2000), soybean phosphatidylcholine (SPC) and cholesterol were purchased from Avanti Polar Lipids Inc. TMC (viscosity 10–50 mPa s, degree of deacetylation 85%, degree of trimethyl substitution 50%) was bought from Golden‐Shell. 1,1′‐Dioctadecyl‐3,3,3′,3′‐tetramethylindocarbocyanine perchlorate (DiI), 2‐(4‐amidinophenyl)‐6‐indolecarbamidinedihydrochloride (DAPI), Lipofectamine 2000, total superoxide dismutase (SOD) assay kit with WST‐8, lipid peroxidation malondialdehyde (MDA) assay kit, glutathione (GSH) assay kit were bought from Beyotime. Cell Counting Kit‐8 (CCK‐8) was obtained from Dojindo. Both human and rat siVEGF and FAM‐labeled siVEGF (siVEGF^FAM^) were obtained from Gene Pharma Co., Ltd. The sense strand of human siVEGF was 5′‐GCAGAUUAUGCGGAUCAAATT‐3′ and the antisense strand was 5′‐UUUGAUCCGCAUAAUCUGCTT‐3′. The sense strand of rat siVEGF was 5′‐CCCAUGAAGUGGUGAAGUUTT‐3′ and the antisense strand was 5′‐AACUUCACCACUUCAUGGGTT‐3′. The primary antibodies of cystine/glutamate exchanger (xCT), glutathione peroxidase (GPX) 4, VEGF, β‐actin, and second antibodies were purchased from Abcam. The ELISA kit was used for analyzing the number of cytokines purchased from Multi Sciences Inc. Hematoxylin–eosin (H&E) staining kit and immunohistochemistry (IHC) kit were obtained from Servicebio Inc. CHCl3 was obtained from Chuandong Chemical Co. Ltd. A Millipore water purification system provided deionized water. All other reagents were of analytic grade.

Human corneal epithelial cells (HCECs) were provided by BeNa Culture Collection (BNCC337876) and cultured with Dulbecco's Modified Eagle Medium (Gibco) medium containing 10% FBS (EVERY GREEN) and 1% penicillin–streptomycin and incubated under a 5% CO_2_ atmosphere at 37°C.

### 2.2 Synthesis of NPs

First, INS‐loaded liposomes were prepared using a reversed‐phase evaporation method. Briefly, an appropriate mass ratio of hybrid lipid (12 mg SPC, 4 mg DSPE‐mPEG2000, and 4 mg cholesterol) was dissolved into 5 mL trichloromethane (CHCl_3_): absolute ether (1:1), 1 mL INS solution (4 mg/mL) in citric–Na_2_HPO_4_ buffer (pH 3.0) was added. The mixture was ultrasonicated in a water bath to form w/o emulsion and then transferred into a 100 mL round‐bottomed flask, which was subsequently evaporated under reduced pressure with a rotating speed of 50 rpm at 30°C for 3 h to remove the organic solvent. Afterward, 4 mL citric acid–Na_2_HPO_4_ buffer (pH 5.6) was added to hydrate the films until a homogeneous dispersion and this mixture was transferred to a 10 mL EP tube. Then, 0.5 mL PFOB was added to the mixture, which was sonicated (55 W, four 3 min) with a sonicator (Sonics & Materials Inc.) in an ice bath, and then centrifugation was performed at 6000 rpm/min for 5 min. Supernatants were removed and sediments were collected and resuspended by phosphate‐buffered saline (PBS; pH 7.4), then stored at 4°C for further use. Subsequently, an aliquot of INS‐lip was mixed with the same volume of TMC (0.5 mg/mL) solution in PBS and then shaken and incubated at 4°C for 1 h to prepare TMC‐INS‐lip (TIL). Finally, siVEGF was loaded by electrostatic adsorption with an optimal ratio to obtain siVEGF‐TMC‐INS‐lip (siVEGF‐TIL). siVEGF‐TMC‐lip (siVEGF‐TL) was made using the same protocol but without INS. Similarly, Empty‐TMC‐lip (TL) was also prepared using the same protocol but with the omission of INS and siVEGF.

### 2.3 Characterization of NPs

The morphology NPs was observed by light microscope transmission electron microscope (TEM) (Hitachi H‐7600). The particle size and zeta potential were measured using a laser particle size analyzer system (Nano, ZS90; Malvern Instrument Ltd). UV–vis–NIR absorption spectrum of INS solution at different concentrations, INS‐lip, TIL, and siVEGF‐TIL were recorded using a UV–vis–NIR spectrophotometer (UV‐3600; Shimadzu) at room temperature. To determine the encapsulation efficiency (EE) and drug loading capacity (DLC), INS‐lip, TIL, and siVEGF‐TIL were centrifuged with an ultracentrifuge at 20,000 × g and 4°C for 30 min. The supernatant was measured for free INS using ultra‐high‐performance liquid chromatography (UHPLC; Shimadzu20) equipped with an ultraviolet (UV) variable wavelength detector and Ultimate XB‐C18 column. For UHPLC measurement, the mobile phase was a mixture of water, acetonitrile, and trifluoroacetic acid with a ratio of 68.5:31.5:0.1. The flow rate was 1 mL/min and the detection wavelength was set at 220 nm. The entrapment efficiency and content were calculated by Equations (1) and (2):

(1) $$\mathrm{EE}\;\left(\%\right)=\text{mass of encapsulated drug}/\text{total mass of drug}\times 100\%,$$

(2) $$\mathrm{DL}\;\left(\%\right)=\text{mass of encapsulated drug}/\text{mass of total liposomes}\times 100\%.$$

Loading siRNA capacity and siRNA protection of NPs: Different amounts of TIL NPs prepared in the light of the above method were added to the siVEGF to make the mixed solution (TIL NPs:siVEGF = 0–9:1) and then incubated for 1 h to fully combine the siVEGF with TIL NPs. All samples were evaluated by electrophoresis on a 2% (wt/vol) agarose gel at 80 mV for 40 min. For nuclease protection, the siVEGF and siVEGF‐TIL NPs were incubated with or without RNase at 37°C for 30 min, followed by halting the nuclease activity at 80°C for 5 min. Next, the siVEGF‐TIL NPs incubated with or without RNase were shaken at 4°C for 2 h. Then, the samples were examined with 2% (wt/vol) agarose gel and the electrophoresis was performed at 80 V for 40 min.

### 2.4 In vitro drug release from NPs

For the determination of INS release from NPs, the INS‐lip, TIL, and siVEGF‐TIL were placed in dialysis bags (MWCO 3.5 kDa) and immersed in 50 mL PBS (0.2 mol/L, pH 7.4), then incubated at 37°C and 100 rpm. At each predetermined time, 200 μL of the sample was drawn and replaced with an equal volume of fresh medium. The content of INS was measured by UHPLC. As for the siVEGF release from siVEGF‐TIL, siVEGF^FAM^‐TIL was placed in dialysis bags (MWCO 50 kDa) and immersed in 10 mL of PBS at 37°C with 100 rpm. At each scheduled time, a 200 μL release medium was withdrawn and supplemented with a 200 μL fresh medium. the amount of siVEGF^FAM^ (*λ*_excitation_/*λ*_emission_ = 480 nm/520 nm) was detected by a microplate reader (BioTek Instruments Inc.).

### 2.5 Cellular uptake of NPs

HCECs cells were seeded in confocal dishes at a density of 1 × 10^4^ cells per well and divided into two groups randomly, including the INS‐lip group and TIL group. After culturing for 24 h, the culture medium was replaced with the medium containing INS‐lip or TIL (all NPs were stained with DiI [*λ*_excitation_/*λ*_emission_ = 549 nm/565 nm]), respectively. After different intervals of incubation, cells were washed to remove noninternalized particles, fixed with 4% formaldehyde, and nuclei were stained with DAPI (*λ*_excitation_/*λ*_emission_ = 364 nm/454 nm). The process of cellular uptake was observed with a confocal laser scanning microscope (CLSM) (Nikon). Furthermore, the quantitative intracellular uptake of INS‐lip and TIL at different intervals was analyzed with flow cytometry (FCM).

### 2.6 Dispersion and retention of NPs on ocular anterior segments

The ocular surface of Sprague Dawley (SD) rats was live monitored using an FLI system (Cri Inc.) to access the retention of fluorescent NPs. SD rats were divided into two groups (*n* = 3 per group, INS‐lip, and siVEGF‐TIL), pentobarbital sodium (3%, 1 mL/kg) was used to sedate rats, then 5 μL NPs containing DiR (*λ*_excitation_/*λ*_emission_ = 748 nm/780 nm) were dropped onto the ocular surface, respectively. Subsequently, the relative fluorescence intensity of NPs staying in the corneas for each preset period was observed and recorded with an in vivo imaging system Spectrum (LB983; Bold).

A similar test for topical delivery of NPs to rat's corneas was performed as follows: rats from the three groups (*n* = 3 per group, INS‐lip, siVEGF‐TIL, and siVEGF^FAM^‐TIL) also were treated with the corresponding NPs, respectively (all NPs were stained with DiI [*λ*_excitation_/*λ*_emission_ = 549 nm/565 nm]) and placed in a dark environment for 4 h. Sequentially, the corneas of each rat were enucleated, followed by fixation with a 10% neutral buffered formalin solution. The tissues were embedded in frozen section media for cryosection and cut into 8 μm sections with a cryostat microtome (CM1860; LEICA). The cryosections were washed with PBS twice to remove the frozen media, followed by staining with DAPI for 30 min. The distribution of NPs in the cornea was observed by fluorescence microscopy (Eclipse Ti‐S; Nikon).

### 2.7 Colocalization and cell transfection of siVEGF‐TIL

HCECs cells were seeded in confocal dishes at 1 × 10^4^ cells/well and cultured for 24 h. After that, the culture medium was replaced with the medium containing siVEGF^FAM^‐TIL NPs (TIL were stained with DiI) and incubated for 3 h. Finally, the coverslips were observed with a CLSM (Nikon). Approximately 1 × 10^4^ HCECs cells/well were seeded in six‐well plates and cultured for 24 h. The siVEGF^FAM^‐TIL was added to each well, separately, followed by incubation for 24 h, with Lipofectamine 2000 as a control. Transfection efficiencies were measured by FCM.

### 2.8 RNA extraction and real‐time polymerase chain reaction

The gene expressions of VEGF were analyzed by real‐time polymerase chain reaction (qRT‐PCR). The designed primers are listed in Table S4. The total RNA was extracted using a Steady Pure Universal RNA Extraction Kit (Accurate Biotechnology, Hunan Co., Ltd.). Approximately 1000 ng of total RNA was extracted. Reverse transcription was performed by Evo M‐MLVRT Kitt (Accurate Biotechnology, Hunan Co., Ltd.). After that, qRT‐PCR was performed using CFX96 Real‐Time System (Bio‐Rad) with SYBR Green Supermix (Accurate Biotechnology, Hunan Co., Ltd.). The relative gene expression levels were calculated by the 2^−∆∆^ *C*_*t*_ method using GAPDH as a control. Each gene was analyzed in triplicate to reduce randomization error.

### 2.9 CCK‐8 assays

HCECs were plated into a 96‐well plate at the density of 5 × 10^3^ cells per well and cultured in incubators for 24 h. The growth medium was replaced by the serum‐free medium containing different concentrations of INS and various NPs (TL, siVEGF‐TL, INS‐lip, TIL, siVEGF‐TIL). After being cultured for 24 h, the cells were washed thrice with PBS, and then freshly prepared CCK‐8 solutions were added to each well. The CCK‐8 was used to detect cell viability in vitro according to the manufacturer's instructions for CCK‐8. Absorbance at 450 nm was measured by a microplate reader (BioTek Instruments Inc.).

### 2.10 In vitro inhibition of oxidative stress, inflammation, and neovascularization by NPs

To investigate the antioxidant stress, anti‐neovascularization, and anti‐inflammatory capacity in vitro, HCECs cells were exposed to H_2_O_2_ (1 mmol/L) for 4 h, followed by the addition of TL, siVEGF‐TL, INS, INS‐lip, TIL, siVEGF‐TIL for another 24 h incubation. The levels of SOD, MDA, GSH, VEGF, CD31, TNF‐α, IL‐6, and MMP‐9 were detected by commercial kits following the specifications provided by the manufacturer precisely. The experiments were carried out in triplicate.

### 2.11 The model of alkali burn of the male SD rat

The animal care and procedures were complied with the Principles of Laboratory Animal Care. It conformed to the standards of the ARVO Statement for the use of animals in Ophthalmic and Vision Research. The Science and Technology Ethics Committee of The Second Affiliated Hospital of the Chongqing Medical University approved this research protocol. Two hundred SD rats (male, 7–8 weeks of age) were obtained from the Animal Experiment Center of Chongqing Medical University (Chongqing, China). All animals weighed between 150 and 200 g were housed at constant temperature (20 ± 1°C) and humidity (50 ± 5%). Their diet consisted of standard rat chow and water ad libitum. The right eyes of SD rats were used and the left eyes served as the normal group. SD rats were anesthetized by an intraperitoneal injection of pentobarbital sodium (3%, 1 mL/kg), and local anesthesia was performed by topical application of 0.5% tetracaine (Bausch & Lomb). A 3 mm diameter filter paper soaked with 1 N sodium hydroxide (NaOH) was placed on the central corneal surface for 40 s followed by thorough rinsing with a large amount of sterile isotonic saline (0.9% sodium chloride [NaCl]) for 1 min, immediately.^31^, ^32^, ^33^ The depth of corneal injury was involving corneal epithelium and superficial stroma which was confirmed by H&E (Figure S1).

### 2.12 Clinical evaluations

After alkali burn, the SD rats were randomized into six groups (PBS, siVEGF‐TL, INS, INS‐lip, TIL, siVEGF‐TIL). Five microliters different reagents were dropped into the right eye twice a day respectively. No treatment for left eyes. To observe the degree of corneal opacity, corneal epithelial repair, and CNV, alkali‐burned corneas were examined by portable slit lamps before and after fluorescein sodium staining every day and photographed on Days 1, 3, 7, and 14. The IOP was measured using a handheld tonometer (iLab tonometer; iCare). Corneal opacity was scored using a scale of 0–4 (Grade 0 = completely clear; Grade 1 = slightly hazy, iris and pupils easily visible; Grade 2 = slightly opaque, iris and pupils still detectable; Grade 3 = opaque, pupils hardly detectable; and Grade 4 = completely opaque with no view of the pupils). The corneal epithelial healing rate was calculated according to the following formula (*k* represents the corneal epithelial healing rate, *S*_0_ represents the 0‐day staining area, and *S*_t_ represents the observed staining area):

(3) $$k=S_{0}-S_{t}\times 100\%.$$

For CNV, the total corneal area and vessel area were manually selected with ImageJ. The CNV area was presented as the percentage with the following formula:

(4) $$\left(\text{vessel}\;\text{area}\;\text{chosen}/\text{total}\;\text{corneal}\;\text{area}\right)\times 100\%.$$

### 2.13 UHPLC–MS metabolomics analysis

On Day 14 after the alkali burn, corneal tissues of the PBS group and INS group were collected (*n* = 8 per group) and 25 mg of each sample was weighed into an EP tube, and 500 μL extract solution (methanol:acetonitrile:water = 2:2:1, with the isotopically labeled internal standard mixture) was added. Then the samples were homogenized at 35 Hz for 4 min and sonicated for 5 min in an ice water bath. The homogenization and sonication cycle was repeated three times. Then the samples were incubated for 1 h at −40°C and centrifuged at 12,000 rpm for 15 min at 4°C. The resulting supernatant was transferred to a fresh glass vial for analysis. The quality control sample was prepared by mixing an equal aliquot of the supernatants from all of the samples. LC–MS/MS analyses were performed using a UHPLC system (Vanquish; Thermo Fisher Scientific) with a UPLC BEH Amide column (2.1 mm × 100 mm, 1.7 μm) coupled to Orbitrap Exploris 120 mass spectrometer (Orbitrap MS; Thermo Fisher Scientific). The mobile phase consisted of 25 mmol/L ammonium acetate and 25 ammonia hydroxide in water(pH 9.75) and acetonitrile. The autosampler temperature was 4°C, and the injection volume was 2 μL. The Orbitrap Exploris 120 mass spectrometer was used for its ability to acquire MS/MS spectra on information‐dependent acquisition (IDA) mode in the control of the acquisition software (Xcalibur; Thermo Fisher Scientific). In this mode, the acquisition software continuously evaluates the full scan MS spectrum. The ESI source conditions were set as follows: sheath gas flow rate as 50 Arb, Aux gas flow rate as 15 Arb, capillary temperature 320°C, full MS resolution as 60,000, MS/MS resolution as 15,000 collision energy as 10/30/60 in NCE mode, spray Voltage as 3.8 kV (positive) or − 3.4 kV (negative), respectively.

### 2.14 Western blot

The total proteins were extracted from five whole corneas per group at 14 days. The Bicinchoninic Acid Protein Assay Kit (Beyotime) was used to determine the concentration of protein. The protein samples were separated at a constant voltage in sodium dodecyl sulfate–polyacrylamide gel electrophoresis and transferred to the Immobilon‐P membrane (IPVH00010; Millipore). Membranes were incubated with QuickBlock Western (Beyotime) for 30 min at 37°C and then immersed in primary antibodies including anti‐β‐actin (1:1000), anti‐VEGF (1:1000), anti‐xCT (1:1000), anti‐GPx4 (1:1000) under 4°C overnight. Subsequently, after being washed with TBS/Tween 20 twice, the membranes were probed with secondary antibodies for 1 h at room temperature, followed by washes with TBS/Tween 20. Finally, these membranes were visualized by the ECL detection system (Bio‐Rad). Gray values were measured to analyze the expression level of target proteins.

### 2.15 Antioxidant stress and anti‐inflammatory activity in vivo

Corneas of each group at 14 days were harvested and the levels of SOD, GSH, and MDA were quantified by commercial kits according to the instructions. Also, the corneal tissues were collected and homogenized with RIPA lysate, followed by centrifugation at 15,000 rpm for 15 min. Then, the levels of Glu, TNF‐α, IL‐6, and MMP‐9 in the supernatant were detected by commercial ELISA kits according to the procedure provided by the manufacturer.

### 2.16 Histological and immunohistochemical analysis

At 14 days after treatment with different reagents, the normal and alkali‐burned corneas were enucleated for histological and immunohistochemical analysis, fixed in 10% buffered formalin, and successively dehydrated in a series of concentrations of ethanol and dimethylbenzene. Afterward, the treated tissues were fixed in paraffin, and tissue slices (thickness 8 mm) were stained with H&E. In addition, the level of CD31 in the corneal tissues was identified using IHC.

### 2.17 Statistical analysis

Statistical analysis was performed by the GraphPad Prism 7 program. Quantitative data were reported as mean ± standard deviation. Two‐group comparisons were conducted using a two‐tailed Student's *t*‐test. One‐way analysis of variance followed by Tukey's multiple comparisons test was used for multigroup comparisons. *p* < 0.05 was considered statistically significant.

## 3 RESULTS AND DISCUSSION

### 3.1 Preparation and characterization of NPs

**FIGURE 1:**
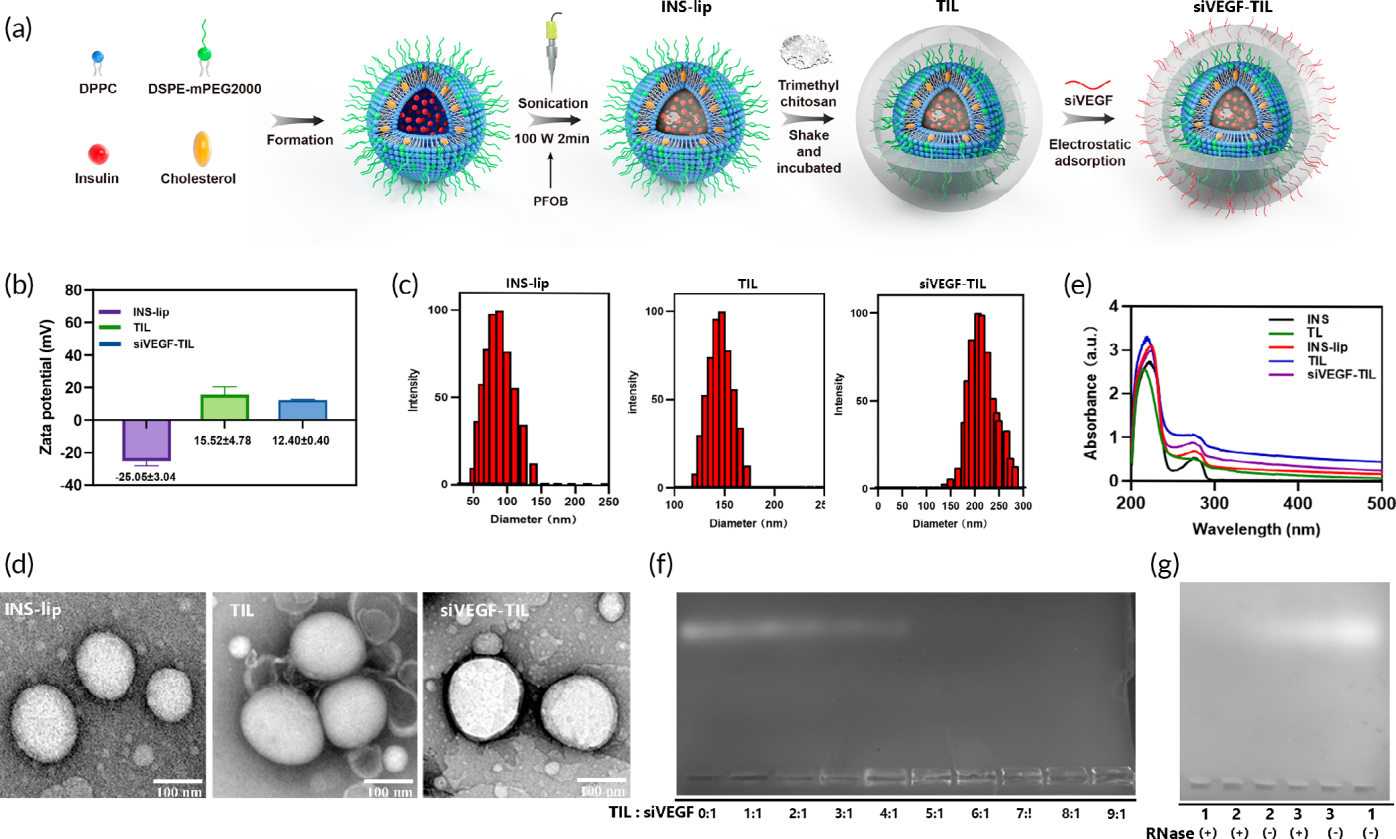
Preparation and characterization of NPs. (a) Schematic illustration of the preparation approach to synthesize siVEGF‐TIL. (b) Zeta potential and (c) size of INS‐lip, TIL, and siVEGF‐TIL (*n* = 3 per group). (d) Transmission electron microscopy (TEM) images of INS‐lip, TIL, and siVEGF‐TIL (scale bar = 100 nm). (e) UV–vis–NIR spectrum of INS, TL, INS‐lip, TIL, and siVEGF‐TIL. (f) Assessment of loading capacity. Agarose gel electrophoresis reflects the amount of unbound siVEGF. With increases in TIL/siVEGF mass ratio, the amount of unbound siVEGF gradually decreased. (g) RNase protection assay in agarose gel electrophoresis. siVEGF‐TIL, siVEGF‐trimethyl chitosan‐coated insulin liposome; INS‐lip, insulin liposome; TIL, trimethyl chitosan‐coated insulin liposome; 1, siVEGF; 2, siVEGF‐TIL; 3, siVEGF‐TIL 2 h (siVEGF‐TIL NPs shook at 4°C for 2 h).

To overcome the physiological and physical barriers of the cornea and improve the bioavailability of drugs, INS was loaded into liposomes (INS‐lip) and coated with TMC to prepare TMC‐coated INS liposomes (TIL). Then, siVEGF was added to optimize the effect of the NPs (siVEGF‐TIL) on the treatment of corneal alkali burns (Figure 1a). To examine the formation of siVEGF‐TIL NPs, zeta potential analysis, particle size analysis, and TEM was performed. INS‐lip could be coated with TMC to prepare TIL via the electrostatic interaction between negative phospholipids and positive TMC, resulting in charge reversal and the enlargement of particle size. Compared with negatively charged INS‐lip (−25.05 ± 3.04 mV), TIL showed a stronger positive charge (+15.52 ± 4.78 mV), and TIL (183.23 ± 12.61 nm) exhibited larger diameters than INS‐lip (124.22 ± 6.04 nm), which indicated that TMC was successfully coated on INS‐lip. As positive NPs, TIL can adsorb negative siRNA; thus, siVEGF‐TIL had a smaller zeta potential (12.40 ± 0.40) and larger diameter (216.63 ± 4.51) than TIL, providing evidence of the loading of siVEGF in TIL (Figure 1b,c; Table S1). TEM images indicated that INS‐lip, TIL, and siVEGF‐TIL had the uniform and spherical appearances; notably, TIL and siVEGF‐TIL were both coated with a transparent TMC shell (Figure 1d).

To demonstrate the feasibility of entrapping INS, UV–vis–NIR spectra were recorded. As Figure 1e shows, INS‐lip, TIL, and siVEGF‐TIL featured the characteristic absorption of INS at 274 nm, suggesting the successful encapsulation of INS into NPs, while TMC‐coated liposomes (TL) had no characteristic INS absorption peaks. EE and DL directly influence treatment efficacy and the administration of NPs. Adequate doses of medication are a prerequisite for effective treatment. The INS EE% values of INS‐lip, TIL, and siVEGF‐TIL were 64.58 ± 2.75%, 65.98 ± 3.96%, and 64.75 ± 6.22%, respectively. The INS DL% of INS‐lip, TIL, and siVEGF‐TIL were 25.83 ± 1.10%, 26.39 ± 1.58%, and 25.90 ± 2.49%, respectively. INS EE% and DL% showed no significant differences among INS‐lip, TIL, and siVEGF‐TIL (*p* > 0.05), which indicated that neither TMC nor siVEGF affected the EE or DL of INS liposomes (Table S1). To confirm the siRNA binding capabilities of TIL, agarose gel electrophoresis was performed after mixing the TIL with siVEGF at different TIL/siRNA ratios. As shown in Figure 1f, the migration of siVEGF in the gel gradually slowed as TIL ratios increased. Almost no free siVEGF could be detected at mass ratios above 5, demonstrating the complete binding of siVEGF by TIL conjugates. The capability of TIL to protect siRNA from nuclease degradation was verified by incubating siVEGF‐TIL with RNase A for 30 min. As shown by agarose gel electrophoresis assays, the naked siVEGF RNase (−) group had a free RNA band, while the naked siVEGF RNase (+) group had no visible bands, indicating that siVEGF had been degraded in the presence of RNase. Regarding siVEGF‐TIL, both the RNase (+) and RNase (−) groups showed no apparent bands, but siVEGF‐TIL NPs shook at 4°C for 2 h with or without RNase both could observe the bands, indicating that siVEGF could be released from the NPs and siVEGF‐TIL could protect siVEGF from RNase degradation (Figure 1g).

### 3.2 Sustained release of INS and siRNA in vitro

**FIGURE 2:**
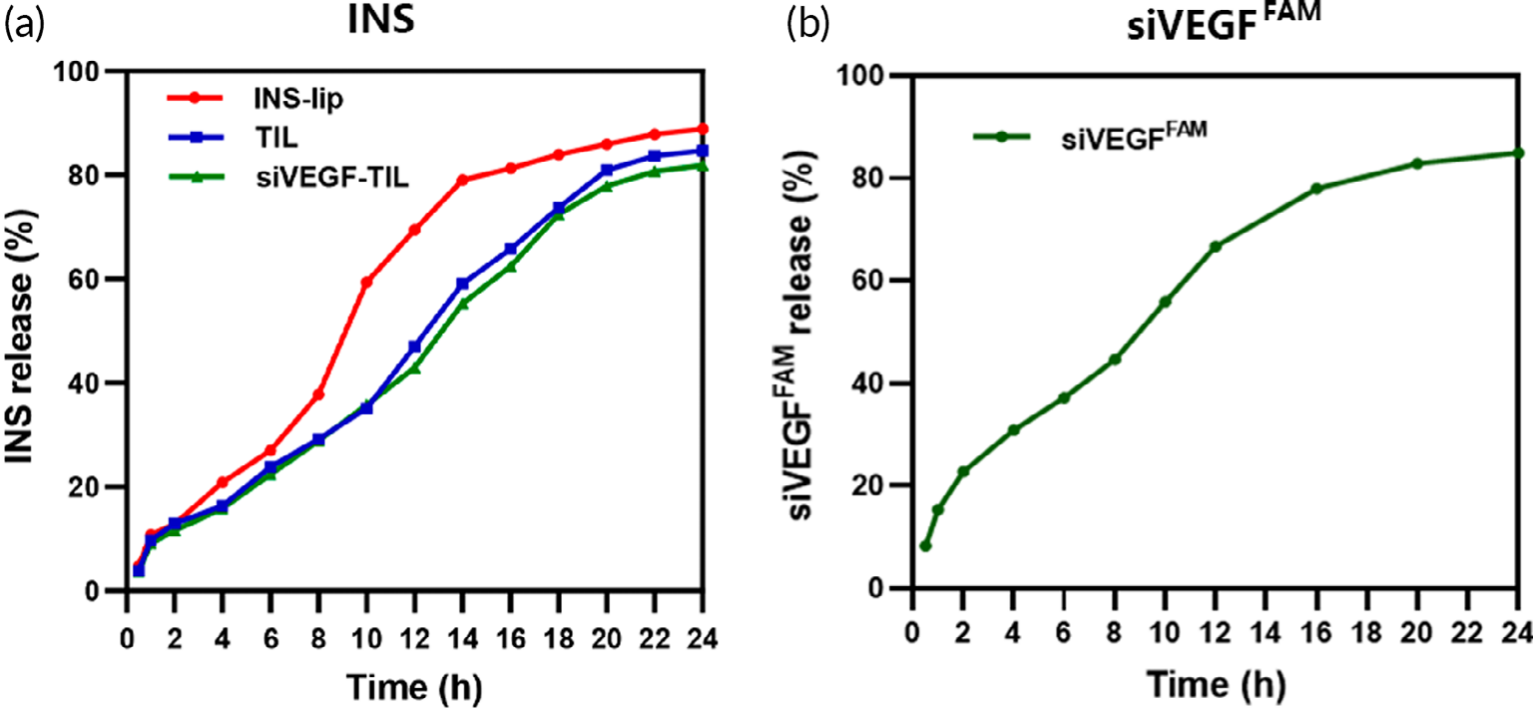
Sustained release of INS and siRNA in vitro. INS release kinetics (a) in INS‐lip, TIL, and siVEGF‐TIL, and siVEGF release kinetics (b) in siVEGFAM‐TIL. INS‐lip, insulin liposome; TIL, trimethyl chitosan‐coated insulin liposome; siVEGF‐TIL, siVEGF‐trimethyl chitosan‐coated insulin liposome.

As shown in Figure 2a, the cumulative release of INS from INS‐lip, TIL, and siVEGF‐TIL was 86.38 ± 3.22%, 85.06 ± 1.93%, and 83.54 ± 1.56%, respectively. In INS‐lip, the cumulative release of INS increased suddenly after 8 h, which might be attributed to the rupture of INS‐lip, contributing to the quick release of drugs. Due to the electronic interactions between TMC and phospholipids, which could promote the stability of the liposomes, the release of INS was slow and sustained in TIL.^34^ At the same time, siVEGF‐TIL and TIL had similar INS release curves and cumulative release, verifying that siVEGF did not affect the release of INS. Furthermore, the siRNA release kinetics of siVEGF‐TIL revealed sustained‐release properties (Figure 2b).

Overall, siVEGF‐TIL and TIL showed ideal sustained release, which was conducive to maintaining concentrations of drugs and genes in the cornea and thus provided potent and prolonged therapeutic efficacy.^35^ In addition, the sustained‐release system can decrease the side effects of drugs on the cornea and significantly improve medication safety.^36^ Furthermore, the sustained release of drugs can reduce dosing frequency, which is one way to enhance patient adherence.^37^

### 3.3 Efficient delivery of NPs in vitro and in vivo

**FIGURE 3:**
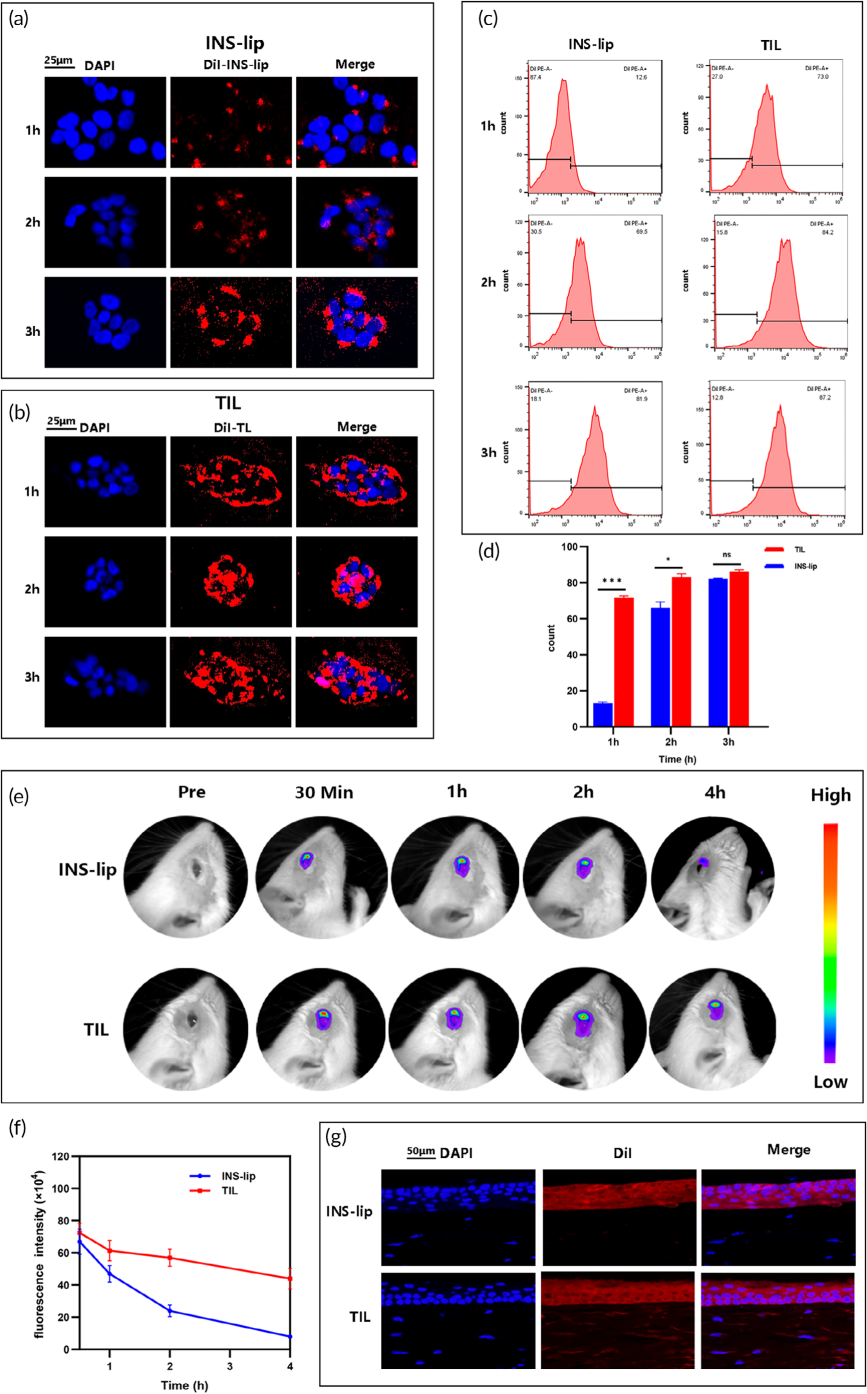
Efficient delivery of NPs in vitro and in vivo. Efficient delivery of NPs in vitro and in vivo. Intracellular uptake of INS‐lip (a) and (b) TIL as observed by CLSM after various intervals of incubation with human corneal epithelial cells. The scale bars are 25 μm. (c) Flow cytometry analysis of intracellular uptake of INS‐lip or TIL labeled with DiI. (d) Quantitative results following flow cytometry analysis of cellular uptake in INS‐lip or TIL groups (*n* = 3 per group). Results were presented as the mean ± SD.*p < 0.05; **p < 0.01; ***p < 0.001. Representative fluorescence images of rat eyes (e) and quantification (*n* = 3 per group) of the fluorescence signal (f) at different time points after topical administration of INS‐lip or TIL. Results were presented as the mean ± SD. (g) DiI‐stained INS‐lip and TIL suspensions were dropped into rat eyes and the permeation capacities of the nanoparticles into corneal tissues were evaluated after 4 h. Scale bar = 50 μm. *n* = 3 per group. CLSM, confocal laser scanning microscope; INS‐lip, insulin liposome; TIL, trimethyl chitosan‐coated insulin liposome.

Efficient intracellular uptake of NPs is required to improve the therapeutic efficacy of drugs.^38^ Therefore, a CLSM was performed to examine the intracellular uptake of NPs in this study. As shown in Figure 3a,b, in HCECs, DiI‐TIL could be phagocytized faster and more effectively than DiI‐INS‐lip. The FCM results (Figures 3c,d) showed more red fluorescence in the cells that were incubated with DiI‐TIL than in cells treated with DiI‐INS‐lip, especially at 1 and 2 h (*p* < 0.05). As shown in Figure 3e,f, fluorescent images were used to evaluate the residence time of NPs on the ocular surface. The attenuation of the fluorescence intensity in the DiR‐INS‐lip group was significantly faster than that in the DiR‐TIL group(*p* < 0.05). Four 4 h later, the fluorescence signal decreased 10‐fold in DiR‐INS‐lip‐treated eyes, whereas it only decreased 2‐fold in DiR‐TIL‐treated eyes, indicating an improved residence time of TIL compared with INS‐lip. Morever, to verify NPs permeation into the tissue, the distribution of the NPs throughout cornea layers in posttreatment corneal cryosections was investigated by fluorescence microscopy. Red fluorescence was detected throughout deep stromal layers in eyes collected 4 h after DiI‐TIL drop application, whereas red fluorescence was observed only in the corneal epithelium in the DiI‐INS‐lip group (Figure 3g). Compared with negatively charged INS‐lip, positively charged TIL could enhance corneal permeability and significantly increase drug transcorneal penetration through strong interactions with the negatively charged corneal surface and the substantial tissue adhesive property of TMC.^39^

### 3.4 Colocalization of siRNA and TIL and the efficacy of siVEGF‐TIL mediated VEGF downregulation

**FIGURE 4:**
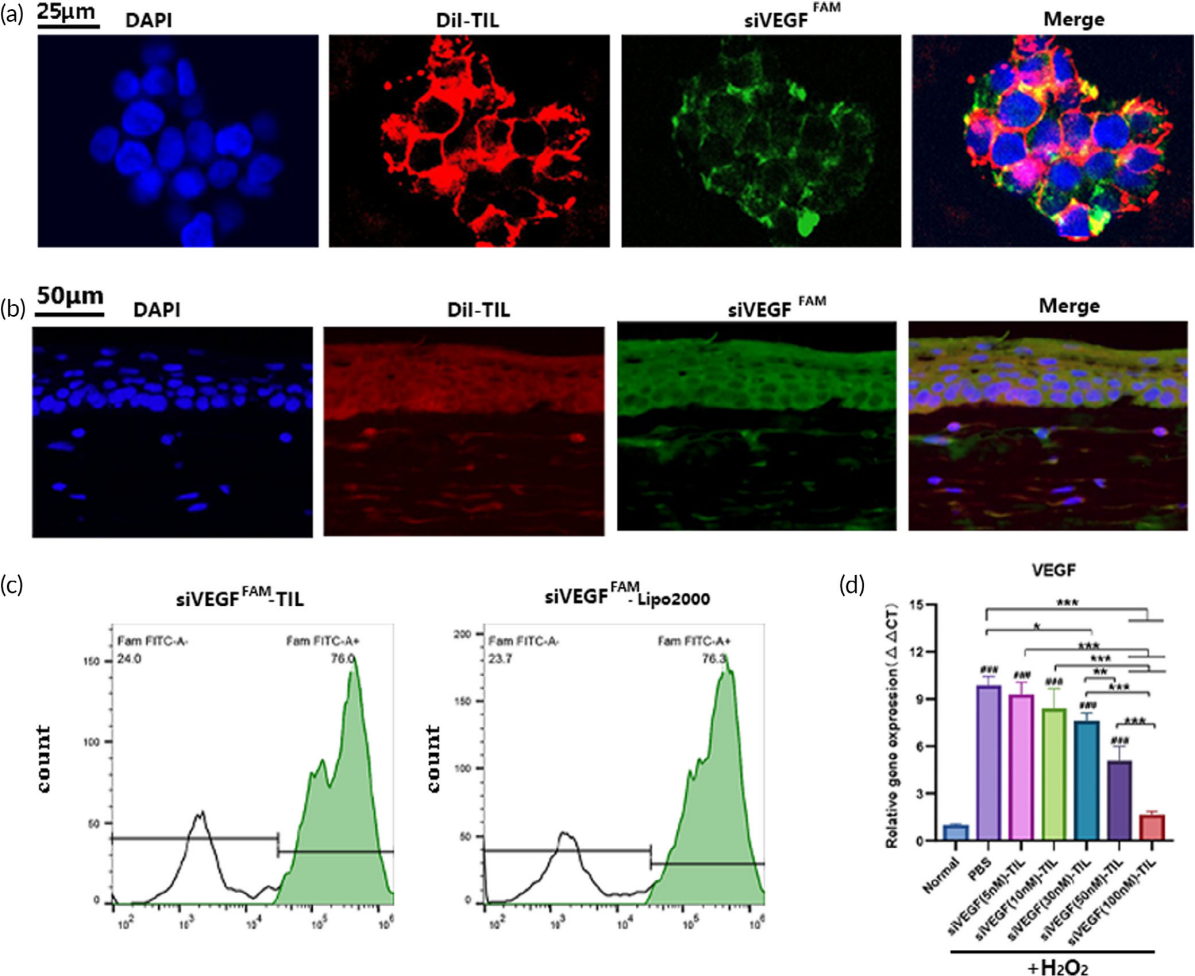
Colocalization of siRNA and TIL and the efficacy of siVEGF‐TIL mediated VEGF downregulation. (a) CLSM images showing the colocalization of TIL labeled with DiI (red) and siVEGF labeled FAM (green) when siVEGF‐TIL was incubated with human corneal epithelial cells for 3 h. The nuclei were stained with DAPI (blue). The scale bars are 25 μm. *n* = 3 per group. (b) siVEGF‐TIL suspensions were dropped into rat eyes and the colocalization of TIL labeled with DiI (red) and siVEGF labeled FAM (green) into corneal tissues was evaluated after 4 h. Scale bars = 50 μm. *n* = 3 per group. (c) Flow cytometry analysis of transfection rates of siVEGF‐TIL and siVEGF‐Lipo2000, the siVEGF was labeled with FAM. Results were presented as the mean ± SD. *n* = 3 per group. (d) The expression of the VEGF gene in cells after treating H_2_O_2_‐HCECs with different content of siVEGF in siVEGF‐TIL for 24 h (*n* = 3 per group). Results were presented as the mean ± SD. *p < 0.05; **p < 0.01; ***p < 0.001. CLSM, confocal laser scanning microscope; siVEGF‐TIL, siVEGF‐trimethyl chitosan‐coated insulin liposome; TIL, trimethyl chitosan‐coated insulin liposome.

As shown in the merged confocal microscopic images (Figure 4a), after 3 h of incubation, the majority of siVEGF^FAM^ overlapped with DiI‐TIL, demonstrating the strong colocalization of siVEGF and TIL in HCECs. CLSM showed that siVEGF^FAM^ was colocalized with DiI‐TIL in corneal cryosections and penetrated the deep stromal layer at 4 h after the administration of eye drops containing siVEGF^FAM^‐DiI‐TIL (Figure 4b).

The transfection efficiency of siVEGF‐TIL was examined by FCM, and siVEGF^FAM^‐Lipofectamine 2000 (siVEGF^FMA^‐Lipo 2000) served as the control. The concentration of siVEGF in the various formulations used was 100 nM. The transfection rate was approximately 63.92 ± 5.36% for siVEGF^FAM^‐TIL and 68.20 ± 5.90% for siVEGF^FMA^‐Lipo2000 (Figures 4c and S2). Compared with siVEGF^FMA^‐Lipo2000, siVEGF‐TIL exhibited almost the same transfection efficiency. These results showed that siVEGF‐TIL had adequate transfection efficiency in vitro.

To identify the efficiency of siVEGF‐TIL in downregulating VEGF expression, qRT‐PCR was performed. As shown in Figure 4d, qRT‐PCR demonstrated that compared with that in the normal group, VEGF expression was increased in all H_2_O_2_‐induced HCECs groups. After treating H_2_O_2_‐HCECs with different levels of siVEGF in siVEGF‐TIL for 24 h, VEGF expression was significantly downregulated in the siVEGF (30 nM)‐TIL, siVEGF (50 nM)‐TIL, and siVEGF (100 nM)‐TIL groups, and higher levels of siVEGF had better effects. There was a 2.06‐fold stronger effect on VEGF downregulation in the siVEGF (100 nM)‐TIL group than the siVEGF (50 nM)‐TIL group, suggesting that siVEGF (100 nM)‐TIL was the best choice to alleviate oxidative stress‐induced increases in VEGF. Therefore, siVEGF (100 nM)‐TIL was selected for subsequent experiments.

### 3.5 NPs improve the viability of H_2_O_2_ ‐stimulated HCECs

As shown in Figure S3A–E, all groups showed good HCECs viability after 24 h of coculture with different concentrations of INS (50–500 μg/mL) or different types of NPs (100–2000 μg/mL), demonstrating good biocompatibility and ensuring the validity of subsequent experiments. The cells in the INS (300 μg/mL), INS‐lip (1000 μg/mL), TIL (1000 μg/mL), and siVEGF‐TIL (1000 μg/mL) groups showed the highest viability (133.35 ± 11.74%, 140.60 ± 8.61%, 143.51 ± 9.12%, and 141.29 ± 15.42%, respectively), therefore these conditions were selected for the further experiments. For consistency with other NPs, TL (1000 μg/mL) and siVEGF‐TL (1000 μg/mL) were selected, and cell viability was 98.15 ± 9.03% and 102.31 ± 11.78%, respectively. The viability of H_2_O_2_‐induced HCECs in the PBS, TL, siVEGF‐TL, INS, INS‐lip, TIL, and siVEGF‐TIL groups was also verified by CCK‐8 assays (Figure S3F). Although H_2_O_2_ significantly decreased cell viability in all groups, it is noteworthy that reagents containing INS could increase cell viability. Among them, the TIL and siVEGF‐TIL groups exhibited the highest cell viability (74.38 ± 8.42% and 73.98 ± 3.47%) compared to the other groups, which showed that TIL and siVEGF‐TIL were the most effective reagents to resist the oxidative stress induced by H_2_O_2_.

### 3.6 In vitro inhibition of oxidative stress, inflammation, and neovascularization by NPs

**FIGURE 5:**
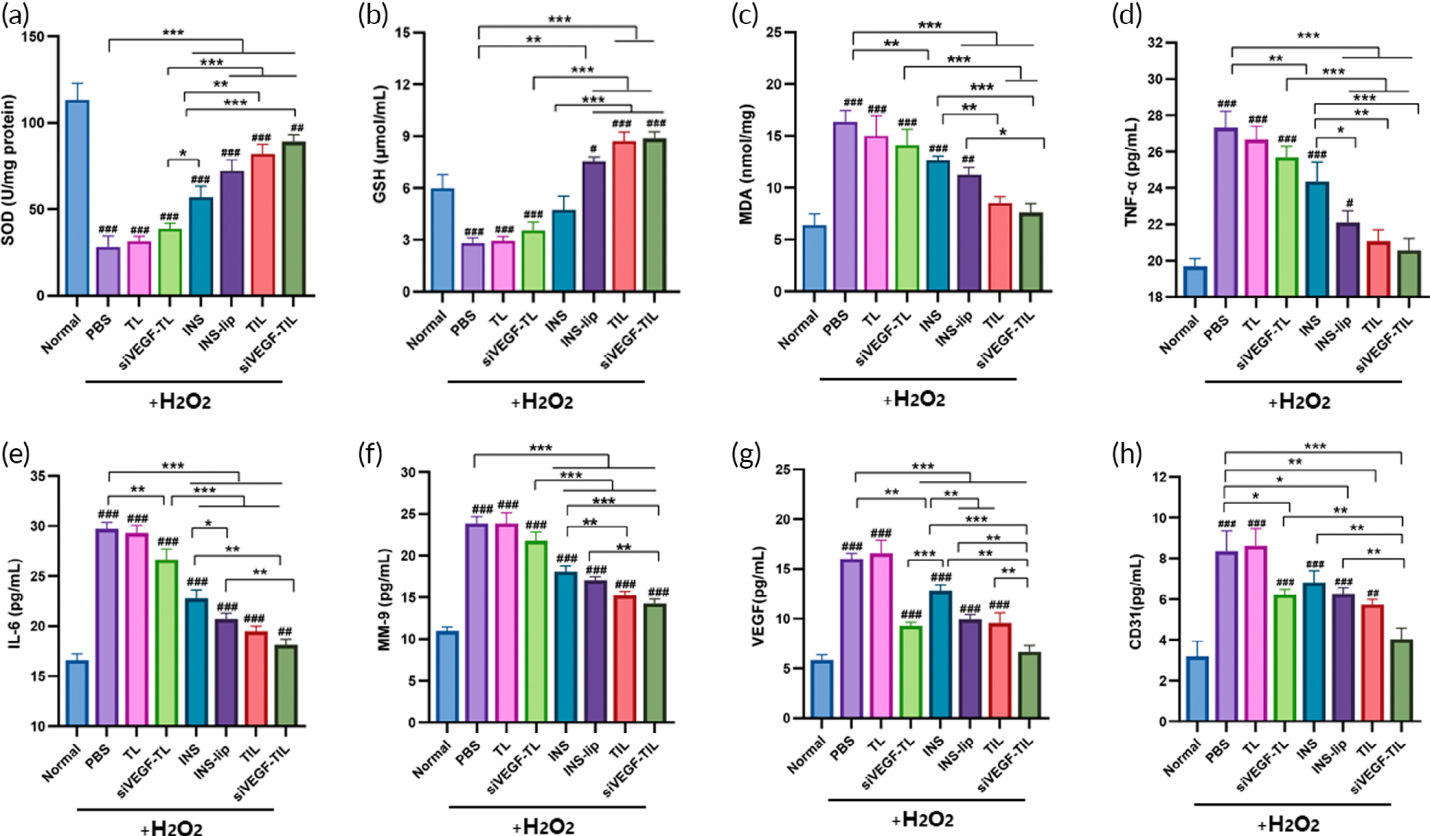
In vitro inhibition of oxidative stress, inflammation, and neovascularization by NPs. SOD activity (a), GSH concentration (b), MDA content (c), TNF‐α (d), IL‐6 (e), MMP‐9 (f), VEGF (g), and CD31 (h) concentrations in H_2_O_2_‐activated human corneal epithelial cells that received different treatments (*n* = 3 per group). Results were presented as the mean ± SD. *p < 0.05; **p < 0.01; ***p < 0.001. Comparison between each group and the normal group (^#^ *p* < 0.05; ^##^ *p* < 0.01; ^###^ *p* < 0.001). GSH, glutathione; INS, insulin; INS‐lip, insulin liposome; MDA, malondialdehyde; siVEGF‐TL, siVEGF‐trimethyl chitosan‐coated liposome; siVEGF‐TIL, siVEGF‐trimethyl chitosan‐coated insulin liposome; SOD, superoxide dismutase; TIL, trimethyl chitosan‐coated insulin liposome; TL, trimethyl chitosan‐coated liposome.

First, the levels of SOD, GSH, and MDA were examined to verify the ability of NPs to inhibit oxidative stress in vitro. Despite decreased SOD and GSH levels in all H_2_O_2_‐induced groups, the INS, INS‐lip, TIL and siVEGF‐TIL groups showed higher levels of SOD and GSH than the PBS group, and this increase was most significant in the TIL and siVEGF‐TIL groups (Figure 5a,b). In contrast, MDA levels were markedly increased in all H_2_O_2_‐induced groups. However, all reagents containing INS alleviated this change in MDA levels, and TIL and siVEGF‐TIL showed the strongest inhibitory effect (Figure 5c).

TNF‐α, IL‐6, and MMP‐9 concentrations in cells were further analyzed to determine the anti‐inflammatory effect of NPs. As shown in Figure 5d–f, the concentrations of TNF‐α, IL‐6, and MMP‐9 in all H_2_O_2_‐induced groups were markedly increased. INS, INS‐lip, TIL, and siVEGF‐TIL treatment reduced the levels of secreted TNF‐α, IL‐6, and MMP‐9. Among them, TIL and siVEGF‐TIL had the strongest anti‐inflammatory effects. These experiments demonstrated that INS and reagents containing INS could suppress oxidative stress and the inflammatory reaction induced by H_2_O_2_, and TIL and siVEGF‐TIL were the most potent.

Next, ELISA was used to examine VEGF and CD31 levels to evaluate the ability of NPs to inhibit neovascularization. The levels of VEGF and CD31 were markedly increased in H_2_O_2_‐induced groups, except the siVEGF‐TIL group. As shown in Figure 5g,h, among the experimental groups, the best therapeutic effect was observed in cells treated with siVEGF‐TIL. VEGF and CD31 levels decreased to 6.70 ± 0.60 and 4.03 ± 0.55 pg/mg protein, respectively. In contrast, weaker suppression of VEGF and CD31 was observed in the TIL and siVEGF‐TL groups than in the siVEGF‐TIL group.

These results showed that although TIL can inhibit oxidative stress and inflammation, it can not effectively resist neovascularization. siVEGF‐TL has a certain inhibitory effect on neovascularization, but its ability to inhibit oxidative stress and inflammation is poor. However, siVEGF‐TIL combines INS and siVEGF, which not only can facilitate HCECs resistance to oxidative stress and the inflammatory response but also inhibit VEGF and CD31 expression in HCECs under oxidative stress.

### 3.7 Clinical evaluation of healing in the corneal alkali burn rat model

**FIGURE 6:**
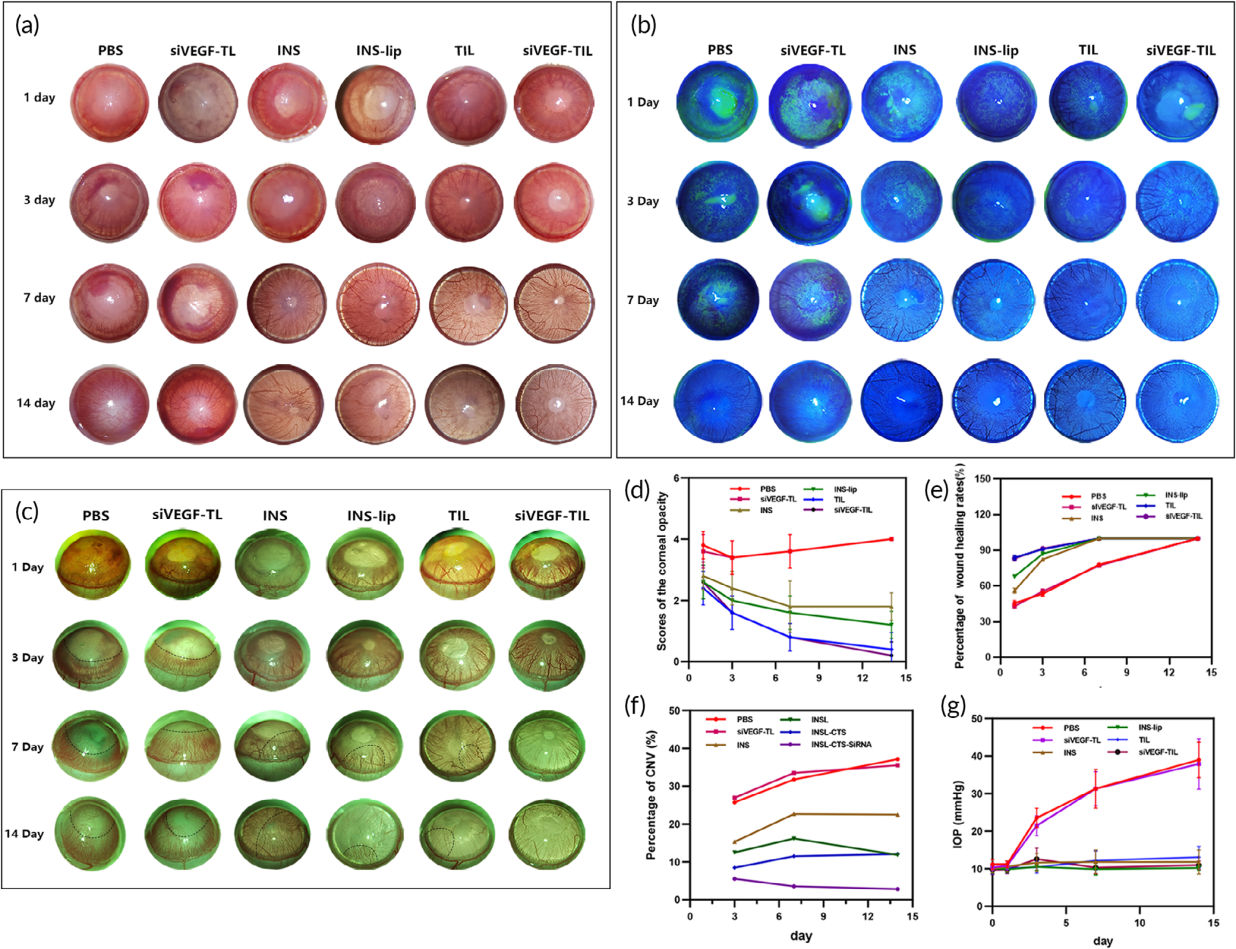
Clinical evaluation of healing in the corneal alkali burn rat model. (a) The anterior views of alkali‐burned eyes were photographed on 1, 3, 7, and 14 days after corneal alkali burn. (b) After the cornea was stained with fluorescein sodium, the corneal epithelium was observed on 1, 3, 7, and 14 days by the portable slit lamps. The green area represents the corneal epithelial defect stained with fluorescein. (c) The side views of alkali‐burned eyes were photographed on 1, 3, 7, and 14 days to observe corneal neovascular outgrowths. (d) Scores of the corneal opacity in each group on Days 1, 3, 7, and 14 (*n* = 5 per group). Results were presented as the mean ± SD. (e) Curves indicated rates of corneal epithelial healing during 1, 3, 7, and 14 days of the treatment (*n* = 5 per group). Data are presented as mean ± SD. (f) The proportion of CNV area in each group on 3, 7, and 14 days (*n* = 5 per group). Data are presented as mean ± SD. (g) Intraocular pressure on Days 0, 1, 3, 7, and 14 in each group. Data are presented as mean ± SD. INS, insulin; INS‐lip, insulin liposome; siVEGF‐TL, siVEGF‐trimethyl chitosan‐coated liposome; siVEGF‐TIL, siVEGF‐trimethyl chitosan‐coated insulin liposome; TIL, trimethyl chitosan‐coated insulin liposome.

Alkali burns were applied to SD rats to produce experimental corneal injury and induce CNV. To assess the therapeutic efficacy of siVEGF‐TIL in vivo on corneal alkali burns, we compared the extent of the burn response and neovascularization in cauterized corneas treated with PBS, siVEGF‐TL, INS, INS‐lip, TIL, and siVEGF‐TL. Each eye drop was topically administered twice per day for 14 consecutive days to SD rats. Representative images showing corneal opacity, epithelial defects, and CNV were obtained using a mobile phone and a portable slit lamp (Kowa) on the 1st, 3rd, 7th, and 14th days after treatment (Figure 6a–c). On the first day, the corneas had similar opacity scores in all groups (*p* > 0.05). On third day, the opacity of the corneas in the INS, INS‐lip, TIL, and siVEGF‐TIL groups began to recover, whereas opacity in the PBS and siVEGF‐TL groups remained the same, and anterior chamber bleeding was observed (Figure 6a). On the seventh day, the opacity of the cornea was further alleviated in all groups, including the PBS and siVEGF‐TL groups. These effects were most pronounced in the TIL and siVEGF‐TIL groups (corneal opacity score 0.8 ± 0.45). Remarkably, corneal opacity scores were significantly increased again in the PBS and siVEGF‐TL groups due to worsened scar tissue and CNV on the 14th day. Furthermore, corneal opacity was not significantly attenuated in the INS group (corneal opacity score 1.8 ± 0.45) compared with that on the seventh day (corneal opacity score 1.8 ± 0.84). In contrast, corneal opacity was reduced in the INS‐lip, TIL, and siVEGF‐TIL groups, and the corneas were almost completely transparent after 14 days of treatment in the TIL and siVEGF‐TIL groups (corneal opacity score 0.4 ± 0.55 and 0.5 ± 0.45, respectively) (Figure 6a,f).

The degree of corneal wound healing was evaluated by fluorescein sodium dripping and the corneal epithelia were photographed on the 1st, 3rd, 7th, and 14th days after the alkali burn. On Day 1, the TIL (16.25 ± 2.01%) and siVEGF‐TIL (15.53 ± 3.08%) groups exhibited minimal corneal area staining with fluorescein. On Day 3, corneal epithelial defects decreased to only punctate defects in the INS‐lip (14.85 ± 2.18%), TIL (8.78 ± 1.02%), and siVEGF‐TIL (8.86 ± 1.25%) groups. However, the defect area was present in nearly 50% of the cornea in the PBS (44.51 ± 3.48%) and siVEGF‐TL groups (47.18 ± 2.88%). Corneas healed faster and recovered completely by Day 7 in the INS, INS‐lip, TIL, and siVEGF‐TIL groups, whereas corneal healing was significantly delayed in the PBS and siVEGF‐TL groups (Figure 6b,e).

CNV did not develop during the first 3 days. On the seventh day, CNV was observed in all groups the except siVEGF‐TIL group. On the 14th day, CNV completely reached the burn area, serious scarring appeared on the cornea in the PBS group, and a small amount of CNV appeared in the INS, INS‐lip, and TIL groups. Conversely, CNV was undetectable in the siVEGF‐TIL group (Figure 6c,f). These results showed that siVEGF‐TIL maintained corneal transparency, accelerated epithelialization, and effectively inhibited CNV.

Alkalis saponify the fatty acids in cell membranes, which results in membrane disruption and dissolution; alkali quickly penetrates through the cornea into the deeper parts of the eye, and hyphema is present in the anterior chamber, followed by increased IOP.^40^ On the other hand, early direct chemical injury can cause tissue shrinkage and disruption of the trabecular meshwork and outflow channels. Subsequent chronic inflammation may lead to synechiae and angle closure, which contribute to secondary increased IOP.^41^ Figure 6g shows that the baseline IOP of the rats did not significantly differ among the groups. Statistically significant differences in IOP were first noted on the third day, from then, the median IOP was significantly increased in the PBS group and the siVEGF‐TL group. On Day 14, the median IOP in the PBS group and the siVEGF‐TL group was 38.99 ± 4.72 and 37.90 ± 6.74 mmHg, respectively, while there was no significant difference in the mean and baseline IOP in the other groups. This result proved that INS, INS‐lip, TIL, and siVEGF‐TIL could effectively alleviate alkali burn‐induced damage and prevent an increase in IOP.

### 3.8 UHPLC–MS metabolomics analysis

The mechanism by which siVEGF inhibits VEGF expression and CNV is currently well understood. Briefly, siVEGF binds with RISC, causing the decomposition of the target mRNA to prevent it from being translated into a functional protein. However, the mechanism by which INS affects corneal alkali burn is unclear and was investigated in this study.^42^

**FIGURE 7:**
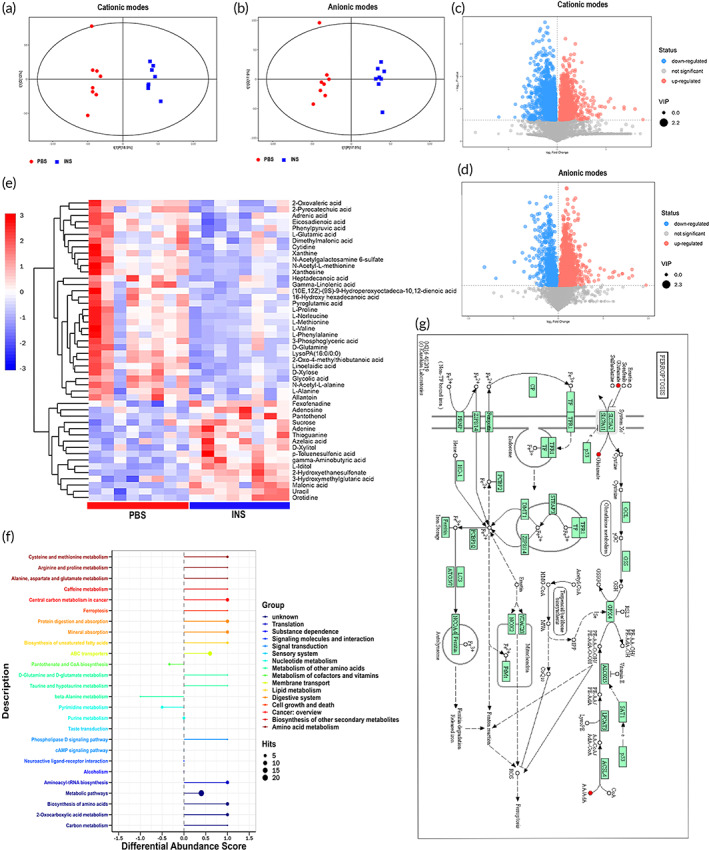
UHPLC–MS metabolomics analysis. OPLS‐DA score plot of the PBS group and INS group under the cationic (a) and anionic mode (b). Volcano plot of the untargeted metabolomics according to the criteria (FC > 1; *p* < 0.05) in cationic (c) and anionic mode (d). compared with the INS group, red stands for upregulated metabolomics, and blue stands for downregulated in the PBS group. (e) Heatmap of the differential metabolites under the anionic mode of untargeted metabolomics. The blue color represents the low relative level of each metabolite, and the red color represents the high relative level of each metabolite. (f) Differential abundance score of KEGG pathway. Different colors indicate that the pathway belongs to different metabolic classifications. The regular value of the line segment indicates that the pathway is upregulated as a whole. On the contrary, it indicates that the pathway is downregulated as a whole. The size of the endpoint of the line segment indicates the amount of material annotated in the path. (g) KEGG pathways plotted of ferroptosis. Red stands for upregulated metabolomics in this pathway in the PBS group, when compared with the INS group. Pathway maps are displayed with copyright permission from KEGG. INS, insulin; OPLS‐DA, orthogonal projection to latent structure‐discriminant analysis.

INS is an anabolic agent; therefore, we hypothesized that INS could treat alkali‐burned corneas, through metabolic regulation. Metabonomics is the accurate metabolomic analysis of dynamic metabolic changes in cells, tissues, and whole organisms.^43^ At 14 days after alkali burns, the corneal tissues of the PBS group and INS group were subjected to UHPLC–MS metabolomics analysis (*n* = 8 per group). Principal component analysis showed a trend in metabolites that were partially separated between the PBS group and INS group, indicating differences among them (Figure S4A,B). To further determine the differences in metabolic profiles between the two groups, orthogonal projection to latent structure‐discriminant analysis (OPLS‐DA) score plots were constructed. As shown in Figure 7a,b, separations in the INS group and PBS group were recognized in both cationic and anionic modes. Then, permutation analysis of the OPLS‐DA model was performed, and the results indicated that the OPLS‐DA model fitting was valid and stable in both ion modes (Figure S4C,D). The altered metabolites were investigated by volcano plot analysis based on the criteria of fold change (FC) > 1 and *p* < 0.05. As shown in Figure 7c,d, red represents upregulated metabolites, and blue represents downregulated metabolites in the PBS group compared with the INS group. Significantly altered metabolites were selected according to VIP > 1 and *p* < 0.05 and were identified by searching the database. Forty‐nine altered metabolites were shown to be significantly different under anionic mode (Figure 7e; Table S2). Based on KEGG analyses, 27 essential signaling pathways associated with these altered metabolites were identified, with 17 associated with significantly higher levels of glutamate in the PBS group than the other group (Table S3).

Glutamate is a nonessential amino acid that naturally occurs in the L‐form and plays an important role in protein and carbohydrate metabolism, boosting resistance to hypoxemia, stimulating oxidation processes, preventing potential redox decreases, affecting glycolysis in tissues, and exerting hepatoprotective effects.^44^ In addition, glutamate is a pivotal regulator of ferroptosis.^45^ In this metabolomic analysis, the ferroptosis pathway was significantly enhanced in the PBS group compared with the INS group, and the differential abundance score was 1 (Figure 7f,g). Ferroptosis is closely associated with oxidative stress. Therefore, we hypothesized that INS and all the INS‐loaded NPs in this study could treat alkali‐burned corneas by decreasing glutamate levels and inhibiting the ferroptosis pathway.

### 3.9 NPs may treat corneal alkali burn by inhibiting the ferroptosis pathway

Ferroptosis is a form of regulated cell death that is driven by peroxidative damage to polyunsaturated fatty acid‐containing phospholipids in cellular membranes. Specifically, ferroptosis is induced by suppressing xCT and GPX4 activity and promoting the accumulation of ROS and a reduction in GSH.^46^ Excessive levels of extracellular glutamate can impair or inhibit cysteine uptake via xCT, resulting in GSH depletion.^45^ GSH depletion decreases GPX4 activity, and lipid peroxides can not be suppressed and metabolized, ultimately accelerating ferroptosis.^47^

**FIGURE 8:**
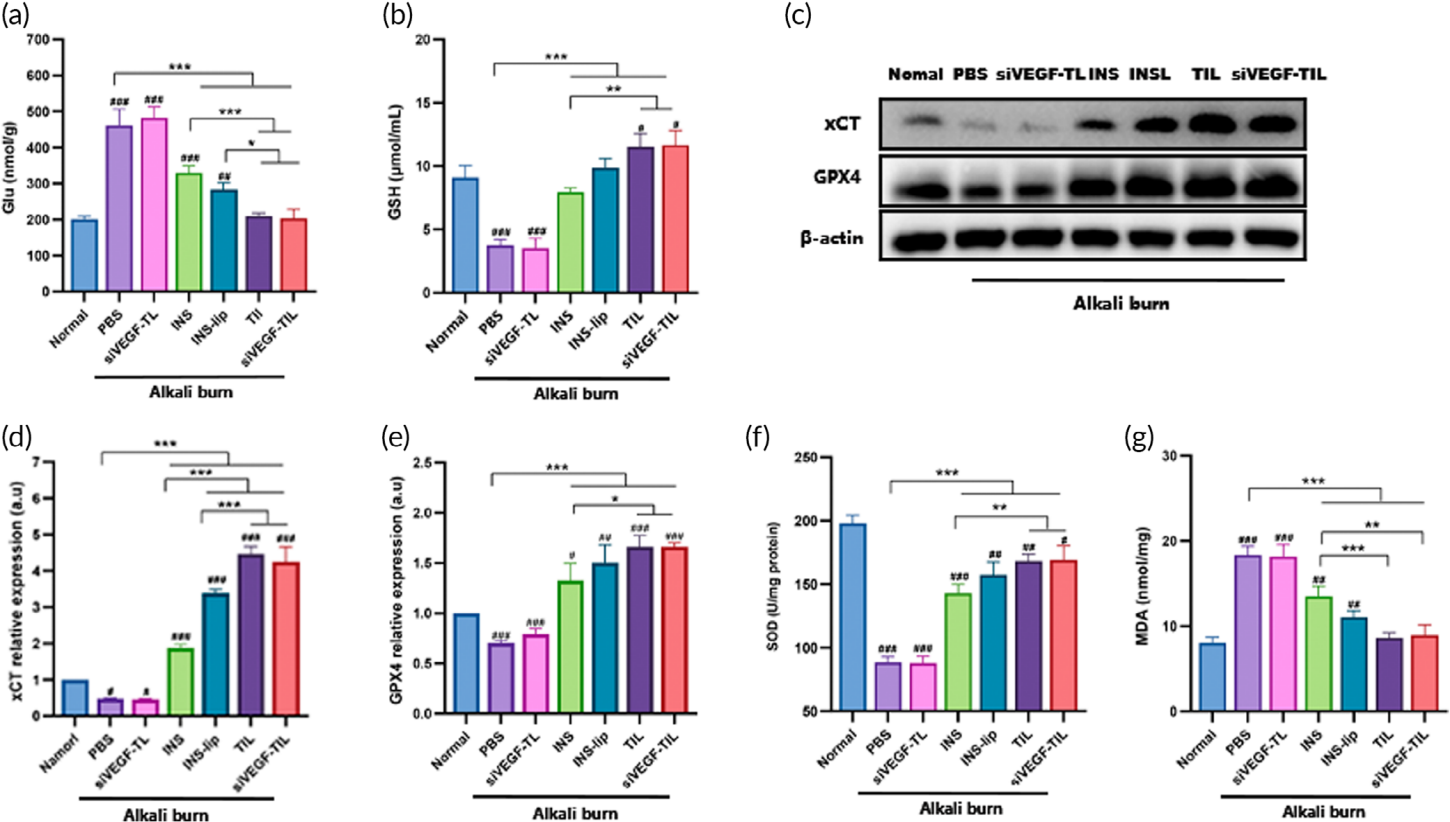
NPs may treat corneal alkali burn by inhibiting the ferroptosis pathway. Glu content (a), and GSH concentration (b) in normal corneas and alkali‐burned corneas that received different treatments (*n* = 3 per group). Results were presented as the mean ± SD. **p < 0.01; ***p < 0.001. Comparison between each group and the normal group (^#^ *p* < 0.05; ^##^ *p* < 0.01; ^###^ *p* < 0.001). (c) Representative Western blots showing xCT and GPX4 in normal corneas and alkali‐burned corneas that received different treatments. The quantification of the Western blot assay for relative expression of xCT (d) and GPX4 (e). Results were presented as the mean ± SD. *p < 0.05; **p < 0.01; ***p < 0.001. Comparison between each group and the normal group (^#^ *p* < 0.05; ^##^ *p* < 0.01; ^###^ *p* < 0.001). *n* = 3per group. SOD activity (f) and MDA content (g) in normal corneas and alkali‐burned corneas that received different treatments (*n* = 3 per group). Results were presented as the mean ± SD. **p < 0.01; ***p < 0.001. Comparison between each group and the normal group (^#^ *p* < 0.05; ^##^ *p* < 0.01; ^###^ *p* < 0.001). Glu, glutamate; GPX4, glutathione peroxidase; GSH, glutathione; INS, insulin; INS‐lip, insulin liposome; MDA, malondialdehyde; siVEGF‐TIL, siVEGF‐trimethyl chitosan‐coated insulin liposome; siVEGF‐TL, siVEGF‐trimethyl chitosan‐coated liposome; SOD, superoxide dismutase; TIL, trimethyl chitosan‐coated insulin liposome; xCT, cystine/glutamate antiporter.

ELISA was performed to compare glutamate levels between each group. The ELISA results showed that glutamate was dramatically increased in all alkali‐burned corneas, but TIL and siVEGF‐TIL treatment could inhibit this outcome (Figure 8a). Next, the levels of GSH in all groups were examined. As shown in Figure 8b, GSH levels in the PBS and siVEGF‐TL groups were significantly decreased. Conversely, when alkali‐burned corneas were treated with INS, INS‐lip, TIL, and siVEGF‐TIL, GSH levels increased significantly, particularly in response to TIL and siVEGF‐TIL treatment. Furthermore, Western blot (WB) analysis was used to measure the protein expressions of xCT and GPX4. The results showed that xCT and GPX4 expression was significantly suppressed in the PBS and siVEGF‐TL groups and was increased in the INS group and various INS‐loaded NPs groups, especially in the TIL group and siVEGF‐TIL group (Figure 8c–e).

We preliminarily confirmed that after alkali damage to the cornea, glutamate in the cornea increased significantly, which activated ferroptosis. INS could inhibit ferroptosis by reducing glutamate levels and activating xCT and GPX4. Liposomal entrapment of INS enhanced this effect. In addition, TIL and siVEGF‐TIL have better adhesion and permeability than other formulations, further improving bioavailability and strengthening the power of the drugs to inhibit glutamate and ferroptosis.

Ferroptosis produces large amounts of ROS, which leads to a severe oxidative stress response. Accordingly, we examined the oxidative stress level in the alkali‐burned corneas of SD rats treated with different reagents. As shown in Figure 8f,g, alkali burn caused a sharp reduction in the level of SOD and an obvious increase in the level of MDA in corneas. After treatment with the various preparations, the MDA concentration was significantly reduced, and the SOD activity had been partially restored in all alkali‐burned corneas compared with those in the PBS group, except for the siVEGF‐TL group. Among the preparations, TIL and siVEGF‐TIL treatment had the most evident effect, demonstrating their superior abilities to inhibit oxidative stress.

### 3.10 In vivo inhibition of inflammation and neovascularization by NPs

**FIGURE 9:**
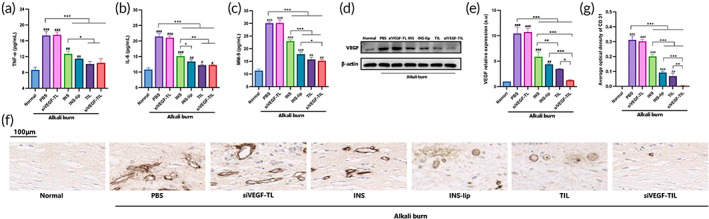
In vivo inhibition of inflammation and neovascularization by NPs. TNF‐α (a), IL‐6 (b), and MMP‐9 (c) concentrations in normal corneas and alkali‐burned corneas that received different treatments (*n* = 3 per group). Results were presented as the mean ± SD. **p < 0.01; ***p < 0.001. Comparison between each group and the normal group (^#^ *p* < 0.05; ^##^ *p* < 0.01; ^###^ *p* < 0.001). (d) VEGF protein expression in normal corneas and alkali‐burned corneas that received different treatments was determined by Western blot. (e) Quantitative analysis of VEGF protein expression in Western blot (*n* = 3 per group). Results were presented as the mean ± SD. **p < 0.01; ***p < 0.001. Comparison between each group and the normal group (^#^ *p* < 0.05; ^##^ *p* < 0.01; ^###^ *p* < 0.001). (f) Immunohistochemical staining of CD31 expression in corneal tissues of each group. Scale bar = 100 μm. (g) Quantitation of CD31 positively stained corneal sections in each group (*n* = 3 per group). Results were presented as the mean ± SD. **p < 0.01; ***p < 0.001. Comparison between each group and the normal group (^#^ *p* < 0.05; ^##^ *p* < 0.01; ^###^ *p* < 0.001). INS, insulin; INS‐lip, insulin liposome; siVEGF‐TIL, siVEGF‐trimethyl chitosan‐coated insulin liposome; siVEGF‐TL, siVEGF‐trimethyl chitosan‐coated liposome; TIL, trimethyl chitosan‐coated insulin liposome.

Corneal alkali burn can lead to oxidative stress and severe inflammatory reactions, which can promote each other. As shown in Figure 9a–c, alkali‐burned corneal tissues showed a sharp increase in the inflammatory cytokines TNF‐α, IL‐6, and MMP‐9. These cytokines were significantly reduced when the corneas were treated with INS, INS‐lip, TIL, and siVEGF‐TIL compared with PBS. Similar to the in vitro results, corneas treated with TIL and siVEGF‐TIL exhibited the largest decrease in cytokine levels.

CNV is a severe complication of corneal injury that not only affects the transparency and avascular nature of the cornea but also makes the cornea more vulnerable to inflammatory reactions.^48^ To verify the ability of NPs to inhibit neovascularization, VEGF protein expression was measured by WB analysis. Compared with that in the PBS group, VEGF expression in corneas in the INS, INS‐lip, TIL, and siVEGF‐TIL groups was reduced, and the strongest anti‐VEGF effect was observed in the siVEGF‐TIL group (Figure 9d,e). In addition, the expression of the endothelial cell‐specific marker CD31 is one of the parameters that could be used to confirm neovascularization. Positive staining for CD31 by IHC was significant in the PBS and siVEGF‐TL groups, and the areas decreased in the INS, INS‐lip, TIL, and siVEGF‐TIL groups; CD31 was hardly expressed in the siVEGF‐TIL group (Figure 9f,g). Interestingly, unlike in the cell experiments, siVEGF‐TL did not inhibit neovascularization in alkali‐burned corneas. This may be because oxidative stress and inflammatory reactions first occur in alkali‐burned corneas, and then they stimulate angiogenic factors and promote neovascularization.^6^ Therefore, inhibiting VEGF without controlling oxidative stress and inflammation does not inhibit CNV. However, siVEGF‐TIL treatment combines the ability of INS to inhibit oxidative stress and inflammation with the ability of siVEGF to inhibit neovascularization; morever, this treatment exhibits superior penetration and adsorption to enhance the bioavailability of drugs and genes, contributing to good therapeutic effects on corneal alkali burns.

### 3.11 Biocompatibility of siVEGF‐TIL in vivo

**FIGURE 10:**
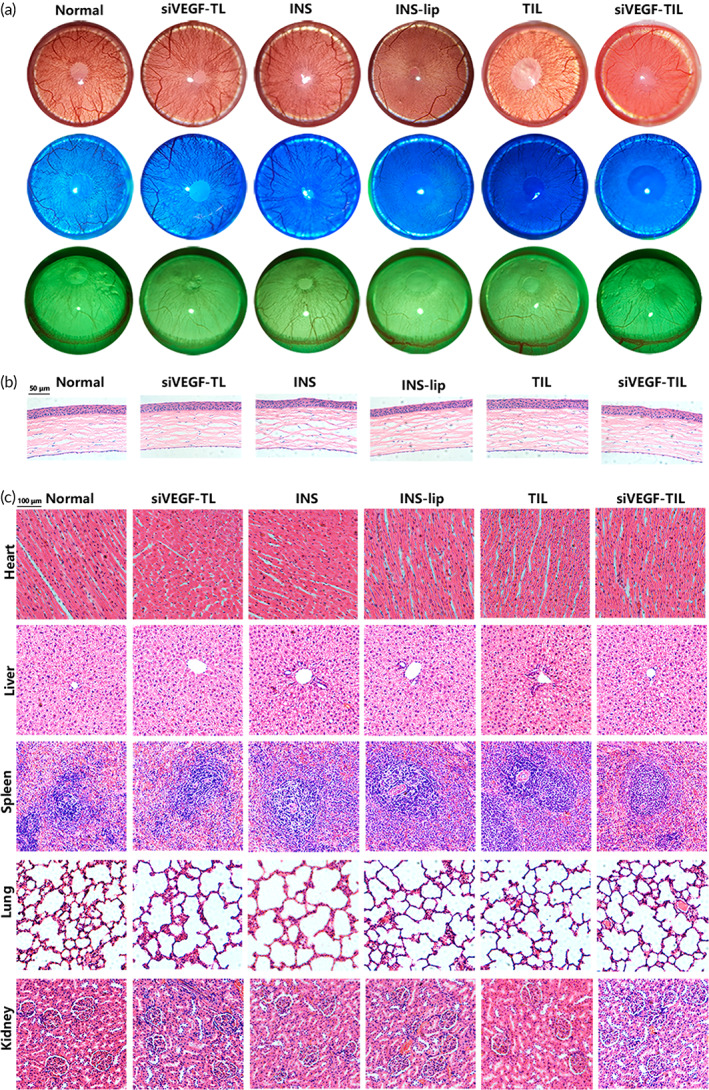
Biocompatibility of siVEGF‐TIL in vivo. After 30 days of dropping different reagents three times a day respectively, in vivo biocompatibility of no administration (healthy) and different formulations treated groups were evaluated by slit‐lamp examination, fluorescein sodium staining (a), and H&E staining (b) on Day 30. Scale bar = 50 μm; *n* = 3. The biocompatibility of different NPs in organs of rats including lung, kidney, liver, heart, and spleen was examined by H&E staining. Scale bar = 100 μm; *n* = 3. INS, insulin; INS‐lip, insulin liposome; siVEGF‐TIL, siVEGF‐trimethyl chitosan‐coated insulin liposome; siVEGF‐TL, siVEGF‐trimethyl chitosan‐coated liposome; TIL, trimethyl chitosan‐coated insulin liposome.

In vivo biocompatibility was assessed by corneal stimulation assessment in normal SD rat eyes treated with the different formulations, followed by corneal examination using a slit‐lamp microscope (Figure 10a). After 30 days of the various treatments, no evidence of corneal opacity, CNV, inflammation, or congestion was found in any corneas. The integrity of the corneal epithelium was evaluated by fluorescein staining. The results showed that the corneal epithelium was intact. In addition, corneal anatomy was examined by H&E staining, and the results showed that the corneas in each group had a regular appearance, were closely and orderly arranged and lacked inflammatory cells or CNV (Figure 10b). Morever, H&E staining of the major visceral organs (heart, liver, spleen, lung, and kidney) revealed that various reagents in this study treatment did not cause significant histological changes. As a result, siVEGF‐TIL NPs have no obvious toxic effects and have excellent biocompatibility, paving the way for clinical applications (Figure 10c).

## 4 CONCLUSION

To the best of our knowledge, this is the first study using a liposome‐TMC nanosystem for the delivery of siVEGF/INS as a combination therapy to treat corneal alkali burns. siVEGF‐TIL treatment showed significant effects in alleviating oxidative stress‐induced HCEC damage (in vitro) and alkali injury in corneas (in vivo). Morever, siVEGF‐TIL treatment had the ideal properties of NPs, including good biosafety profiles, lack of toxicity, facile preparation, adherence, and sustained release, suggesting that this strategy holds potential as a novel delivery platform for the cornea. Furthermore, the molecular mechanism of siVEGF‐TIL treatment was revealed in this study. We found that corneal alkali burn was linked to the regulation of ferroptosis, which could be suppressed by INS. This is also the first report showing the effects of INS on ferroptosis. Notably, siVEGF‐TIL could substantially inhibit both ferroptosis and CNV, eventually preventing alkali damage in corneas. siVEGF‐TIL treatment is an up‐and‐coming therapeutic agent for future clinical applications in corneal damage. There were still many shortcomings in this study. Only male rats were used in this study because males were more susceptible than females to corneal alkali burn. This research did not compare siVEGF TIL NPs with existing treatments for corneal alkali burns (such as topical corticosteroids and NSAIDs). The absolute concentration of INS or siVEFG in the NPs was not detected, which was a limitation of this study regarding the further clinical translation of siVEGF‐TIL treatment.

## AUTHOR CONTRIBUTIONS

**Xiaojing Xiong:** Conceptualization (equal); data curation (equal); formal analysis (equal); investigation (equal); methodology (equal); software (equal); visualization (equal); writing – original draft (equal); writing – review and editing (equal). **Huiting Jiang:** Investigation (equal); methodology (equal); software (equal); validation (equal); visualization (equal). **Yukun Liao:** Investigation (equal); methodology (equal); software (equal); validation (equal). **Yangrui Du:** Project administration (equal); validation (equal); visualization (equal). **Yu Zhang:** Conceptualization (equal); investigation (equal); project administration (equal); validation (equal). **Zhigang Wang:** Methodology (equal); resources (equal); validation (equal); writing – review and editing (equal). **Minming Zheng:** Investigation (equal); methodology (equal); writing – review and editing (equal). **Zhiyu Du:** Conceptualization (equal); formal analysis (equal); funding acquisition (equal); investigation (equal); project administration (equal); writing – review and editing (equal).

## CONFLICT OF INTEREST STATEMENT

The author declare no conflict of interest.

### PEER REVIEW

The peer review history for this article is available at https://publons.com/publon/10.1002/btm2.10499.

## Supporting information


**Data S1.** Supporting Information

## Data Availability

Data available on request from the authors.

## References

[1] Glaudo M , Panfil C , Schrage NF . Defining corneal chemical burns: a novel exact and adjustable ocular model. Toxicol Rep. 2021;8:1200‐1206. 34189056 10.1016/j.toxrep.2021.06.005 PMC8215138

[2] Kethiri A , Singh V , Damala M , et al. Long‐term observation of ocular surface alkali burn in rabbit models: quantitative analysis of corneal haze, vascularity, and self‐recovery. Exp Eye Res. 2021;205:108526. 33662355 10.1016/j.exer.2021.108526

[3] Liu A , Liang C , Liu J , Huang Y , Wang M , Wang L . Reactive oxygen species–responsive lipid nanoparticles for effective RNAi and corneal neovascularization therapy. ACS Appl Mater Interfaces. 2022;14(15):17022‐17031. 35380773 10.1021/acsami.1c23412

[4] Tsai IL , Tsai CY , Kuo LL , Woung LC , Cheng YH . PLGA nanoparticles containing Lingzhi extracts rescue corneal epithelial cells from oxidative damage. Exp Eye Res. 2021;206:108539. 33741324 10.1016/j.exer.2021.108539

[5] Cejka C , Kossl J , Holan V , Zhang J , Cejkova J . An Immunohistochemical study of the increase in antioxidant capacity of corneal epithelial cells by molecular hydrogen, leading to the suppression of alkali‐induced oxidative stress. Oxid Med Cell Longev. 2020;2020:7435260. 32655773 10.1155/2020/7435260 PMC7327556

[6] Zhang K , Guo M , Li Q , et al. Drp1‐dependent mitochondrial fission mediates corneal injury induced by alkali burn. Free Radic Biol Med. 2021;176:149‐161. 34562609 10.1016/j.freeradbiomed.2021.09.019

[7] Roshandel D , Eslani M , Baradaran‐Rafii A , et al. Current and emerging therapies for corneal neovascularization. Ocul Surf. 2018;16(4):398‐414. 29908870 10.1016/j.jtos.2018.06.004 PMC6461401

[8] Mohan R , Kempuraj D , D'Souza S , Ghosh A . Corneal stromal repair and regeneration. Prog Retin Eye Res. 2022;91:101090. 35649962 10.1016/j.preteyeres.2022.101090 PMC11926992

[9] Vercammen H , Miron A , Oellerich S , et al. Corneal endothelial wound healing: understanding the regenerative capacity of the innermost layer of the cornea. Transl Res. 2022;248:111‐127. 35609782 10.1016/j.trsl.2022.05.003

[10] Ramalingam M , Kim SJ . Insulin on hydrogen peroxide‐induced oxidative stress involves ROS/Ca^2+^ and Akt/Bcl‐2 signaling pathways. Free Radic Res. 2014;48(3):347‐356. 24286466 10.3109/10715762.2013.869588

[11] Rajasekar N , Nath C , Hanif K , Shukla R . Intranasal insulin exerts beneficial effects by improving cerebral blood flow, Nrf‐2 expression and BDNF in STZ (ICV) induced memory‐impaired rats. Life Sci. 2016;173:1‐10. 27693383 10.1016/j.lfs.2016.09.020

[12] Yang S , Zhang Y , Zhang Z , et al. Insulin promotes corneal nerve repair and wound healing in type 1 diabetic mice by enhancing Wnt/β‐catenin signaling. Am J Pathol. 2020;190(11):2237‐2250. 32858016 10.1016/j.ajpath.2020.08.006

[13] Cruz‐Cazarim E , Cazarim MS , Ogunjimi AT , Petrilli R , Rocha EM , Lopez R . Prospective insulin‐based ophthalmic delivery systems for the treatment of dry eye syndrome and corneal injuries. Eur J Pharm Biopharm. 2019;140:1‐10. 31015020 10.1016/j.ejpb.2019.04.014

[14] Diaz‐Valle D , Burgos‐Blasco B , Rego‐Lorca D , et al. Comparison of the efficacy of topical insulin with autologous serum eye drops in persistent epithelial defects of the cornea. Acta Ophthalmol. 2022;100(4):e912‐e919. 34407296 10.1111/aos.14997

[15] Giannaccare G , Pellegrini M , Bovone C , et al. Anti‐VEGF treatment in corneal diseases. Curr Drug Targets. 2020;21(12):1159‐1180. 32189591 10.2174/1389450121666200319111710

[16] Nicholas M , Mysore N . Corneal neovascularization. Exp Eye Res. 2021;202:108363. 33221371 10.1016/j.exer.2020.108363

[17] Zorzi GK , Schuh RS , Campos AMD , Carvalho ELS , Teixeira HF . Biomateriais para formulaes de base nanotecnolgica visando terapia gentica ocular. Química Nova. 2017;40:74‐84.

[18] Supe S , Upadhya A , Singh K . Role of small interfering RNA (siRNA) in targeting ocular neovascularization: a review. Exp Eye Res. 2021;202:108329. 33198953 10.1016/j.exer.2020.108329

[19] Zahir‐Jouzdani F , Soleimani M , Mahbod M , et al. Corneal chemical burn treatment through a delivery system consisting of TGF‐β siRNA: in vitro and in vivo. Drug Deliv Transl Res. 2018;8(5):1127‐1138. 29869292 10.1007/s13346-018-0546-0

[20] Zorzi G , Schuh R , Maschio V , Brazil N , Rott M , Teixeira H . Box Behnken design of siRNA‐loaded liposomes for the treatment of a murine model of ocular keratitis caused by *Acanthamoeba* . Colloids Surf. B. 2019;173:725‐732. 10.1016/j.colsurfb.2018.10.044 30384269

[21] Baran‐Rachwalska P , Torabi‐Pour N , Sutera F , et al. Topical siRNA delivery to the cornea and anterior eye by hybrid silicon‐lipid nanoparticles. J Control Release. 2020;326:192‐202. 32653503 10.1016/j.jconrel.2020.07.004

[22] Er S , Laraib U , Arshad R , et al. Amino acids, peptides, and proteins: implications for nanotechnological applications in biosensing and drug/gene delivery. Nanomaterials. 2021;11(11):3002. 34835766 10.3390/nano11113002 PMC8622868

[23] Sahu B , Chug I , Khanna H . The ocular gene delivery landscape. Biomolecules. 2021;11(8):1135. 34439800 10.3390/biom11081135 PMC8394578

[24] Lu W , Yao J , Zhu X , Qi Y . Nanomedicines: redefining traditional medicine. Biomed Pharmacother. 2021;134:111103. 33338747 10.1016/j.biopha.2020.111103

[25] Wong C , Al‐Salami H , Dass C . Recent advancements in oral administration of insulin‐loaded liposomal drug delivery systems for diabetes mellitus. Int J Pharm. 2018;549:201‐217. 30071309 10.1016/j.ijpharm.2018.07.041

[26] Grigoras A . Polymer‐lipid hybrid systems used as carriers for insulin delivery. Nanomedicine. 2017;13(8):2425‐2437. 28821465 10.1016/j.nano.2017.08.005

[27] Hamedi H , Moradi S , Hudson S , Tonelli A , King M . Chitosan based bioadhesives for biomedical applications: a review. Carbohydr Polym. 2022;282:119100. 35123739 10.1016/j.carbpol.2022.119100

[28] Dai X , Zhao X , Liu Y , et al. Controlled synthesis and surface engineering of Janus chitosan‐gold nanoparticles for photoacoustic imaging‐guided synergistic gene/photothermal therapy. Small. 2021;17(11):e2006004. 33619841 10.1002/smll.202006004

[29] Gao Y , Wu Y . Recent advances of chitosan‐based nanoparticles for biomedical and biotechnological applications. Int J Biol Macromol. 2022;203:379‐388. 35104473 10.1016/j.ijbiomac.2022.01.162

[30] Kulkarni A , Patel H , Surana S , Vanjari Y , Belgamwar V , Pardeshi C . *N*,*N*,*N*‐Trimethyl chitosan: an advanced polymer with myriad of opportunities in nanomedicine. Carbohydr Polym. 2017;157:875‐902. 27988003 10.1016/j.carbpol.2016.10.041

[31] Saika S , Ikeda K , Yamanaka O , et al. Expression of Smad7 in mouse eyes accelerates healing of corneal tissue after exposure to alkali. Am J Pathol. 2005;166(5):1405‐1418. 15855641 10.1016/S0002-9440(10)62358-9 PMC1606395

[32] Poon M , Yan L , Jiang D , et al. Inhibition of RAP1 enhances corneal recovery following alkali injury. Invest Ophthalmol Vis Sci. 2015;56(2):711‐721. 25574050 10.1167/iovs.14-15268

[33] Jiang D , Gao F , Zhang Y , et al. Mitochondrial transfer of mesenchymal stem cells effectively protects corneal epithelial cells from mitochondrial damage. Cell Death Dis. 2016;7(11):e2467. 27831562 10.1038/cddis.2016.358 PMC5260876

[34] Kumar S , Dutta J , Dutta P , Koh J . A systematic study on chitosan‐liposome based systems for biomedical applications. Int J Biol Macromol. 2020;160:470‐481. 32464212 10.1016/j.ijbiomac.2020.05.192

[35] Gholizadeh S , Wang Z , Chen X , Dana R , Annabi N . Advanced nanodelivery platforms for topical ophthalmic drug delivery. Drug Discov Today. 2021;26(6):1437‐1449. 33689858 10.1016/j.drudis.2021.02.027

[36] Mofidfar M , Abdi B , Ahadian S , et al. Drug delivery to the anterior segment of the eye: a review of current and future treatment strategies. Int J Pharm. 2021;607:120924. 34324989 10.1016/j.ijpharm.2021.120924 PMC8579814

[37] Mazet R , Yaméogo J , Wouessidjewe D , Choisnard L , Gèze A . Recent advances in the design of topical ophthalmic delivery systems in the treatment of ocular surface inflammation and their biopharmaceutical evaluation. Pharmaceutics. 2020;12(6):570. 32575411 10.3390/pharmaceutics12060570 PMC7356360

[38] Sousa de Almeida M , Susnik E , Drasler B , Taladriz‐Blanco P , Petri‐Fink A , Rothen‐Rutishauser B . Understanding nanoparticle endocytosis to improve targeting strategies in nanomedicine. Chem Soc Rev. 2021;50(9):5397‐5434. 33666625 10.1039/d0cs01127d PMC8111542

[39] Li J , Jin X , Yang Y , Zhang L , Liu R , Li Z . Trimethyl chitosan nanoparticles for ocular baicalein delivery: preparation, optimization, in vitro evaluation, in vivo pharmacokinetic study and molecular dynamics simulation. Int J Biol Macromol. 2020;156:749‐761. 32320806 10.1016/j.ijbiomac.2020.04.115

[40] Lin M , Ekşioğlu Ü , Mudumbai R , Slabaugh M , Chen P . Glaucoma in patients with ocular chemical burns. Am J Ophthalmol. 2012;154(3):481‐485.e1. 22633350 10.1016/j.ajo.2012.03.026

[41] Dohlman C , Zhou C , Lei F , et al. Glaucoma after corneal trauma or surgery—a rapid, inflammatory, IOP‐independent pathway. Cornea. 2019;38(12):1589‐1594. 31453878 10.1097/ICO.0000000000002106 PMC6830965

[42] Ryoo N , Lee J , Lee H , et al. Therapeutic effects of a novel siRNA‐based anti‐VEGF (siVEGF) nanoball for the treatment of choroidal neovascularization. Nanoscale. 2017;9(40):15461‐15469. 28976519 10.1039/c7nr03142d

[43] Gika H , Virgiliou C , Theodoridis G , Plumb R , Wilson I . Untargeted LC/MS‐based metabolic phenotyping (metabonomics/metabolomics): the state of the art. J Chromatogr B Analyt Technol Biomed Life Sci. 2019;1117:136‐147. 10.1016/j.jchromb.2019.04.009 31009899

[44] Salyha N , Salyha Y . Protective role of l‐glutamic acid and l‐cysteine in mitigation the chlorpyrifos‐induced oxidative stress in rats. Environ Toxicol Pharmacol. 2018;64:155‐163. 30412861 10.1016/j.etap.2018.10.010

[45] Li Z , Li Y , Yang Y , Gong Z , Zhu H , Qian Y . In vivo tracking cystine/glutamate antiporter‐mediated cysteine/cystine pool under ferroptosis. Anal Chim Acta. 2020;1125:66‐75. 32674782 10.1016/j.aca.2020.05.049

[46] Lei G , Mao C , Yan Y , Zhuang L , Gan B . Ferroptosis, radiotherapy, and combination therapeutic strategies. Protein Cell. 2021;12(11):836‐857. 33891303 10.1007/s13238-021-00841-y PMC8563889

[47] Li Y , Feng D , Wang Z , et al. Ischemia‐induced ACSL4 activation contributes to ferroptosis‐mediated tissue injury in intestinal ischemia/reperfusion. Cell Death Differ. 2019;26:2284‐2299. 30737476 10.1038/s41418-019-0299-4 PMC6889315

[48] Wang J , Tseng C , Lin F , et al. Topical application of TAK1 inhibitor encapsulated by gelatin particle alleviates corneal neovascularization. Theranostics. 2022;12(2):657‐674. 34976206 10.7150/thno.65098 PMC8692906

