# Supplementary material for: Liposome–trimethyl chitosan nanoparticles codeliver insulin and siVEGF to treat corneal alkali burns by inhibiting ferroptosis
Source: Bioeng Transl Med. 2023 Feb 9;8(2):e10499. doi: 10.1002/btm2.10499 (PMC10013822; doi:10.1002/btm2.10499)
Supplement: Supplementary file 1 — Data S1. Supporting Information [file BTM2-8-e10499-s001.docx]

**Table S1.** Mean size, polydispersity index (PDI), zeta potential, encapsulation efficiency (EE), and drug-loading capacity (DLC) of nanoparticles (n=3).

| Nanoparticles | Size (nm) | PDI | Zeta potential (mV) | EE (%) | DLC (%) |
| --- | --- | --- | --- | --- | --- |
| INS-lip | 96.22±6.04 | 0.25±0.08 | -25.05±3.04 | 67.89±2.04 | 30.55±0.92 |
| TIL | 183.23±12.61 | 0.22±0.01 | 36.88±6.56 | 67.90±6.43 | 30.55±2.90 |
| SiVEGF-TIL | 216.63±4.51 | 0.19±0.02 | 27.12±8.90 | 69.08±3.10 | 31.09±1.39 |

PDI： polydispersity index; INS-lip: insulin liposome; TIL: trimethyl chitosan coated insulin liposome; siVEGF-TIL: siVEGF-trimethyl chitosan coated insulin liposome.


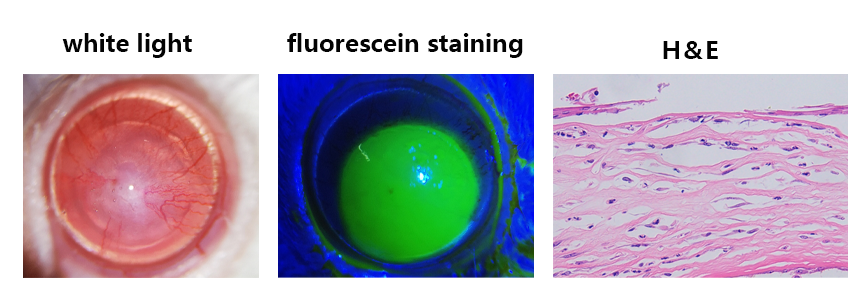


**Figure S1** Slit-lamp pictures and H＆E of SD rats corneal alkali burn.

**Figure S2**. Quantitative results following flow cytometry analysis of transfection rates of siVEGF-TIL and siVEGF-Lipo2000 (n=3 per group). Results were presented as the mean ± SD. siVEGF-TIL: siVEGF-trimethyl chitosan coated insulin liposome.


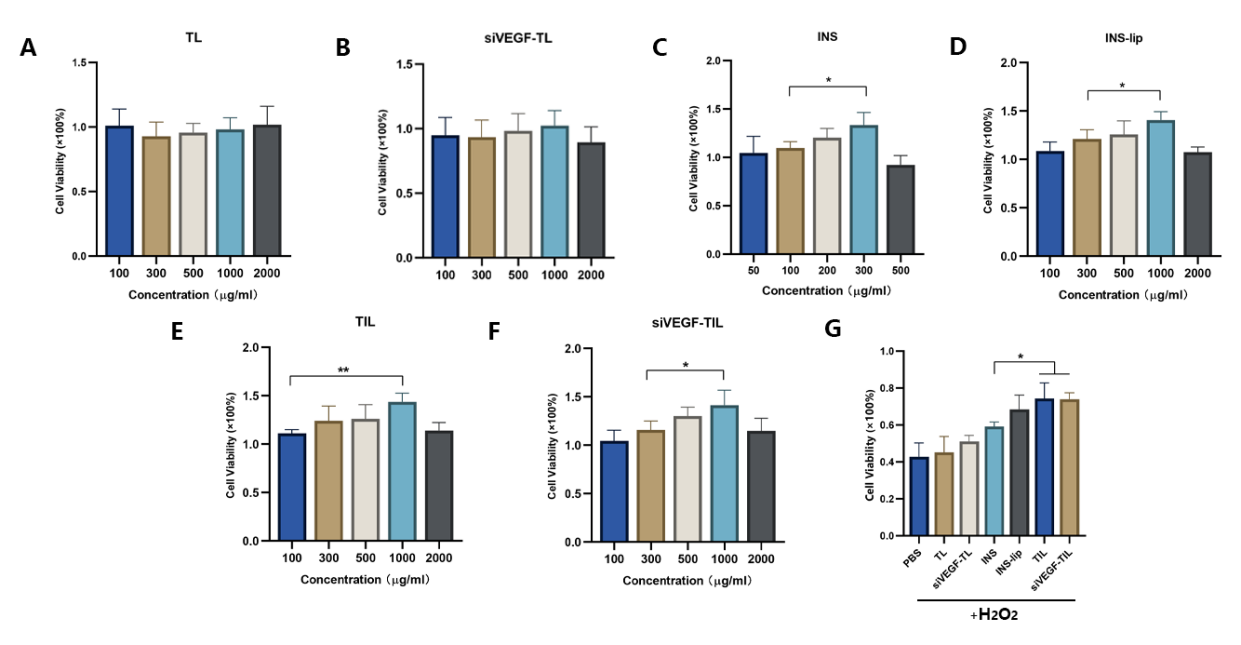


**Figure S3.** The CCK-8 assay of the human corneal epithelial cells treated with different concentrations of TL (A), INS (B), INS-lip (C), TILI(D), and siVEGF-TIL(E) was used to measure cell viability in vitro. Data are presented as mean ± SD (n = 3 per group). **p* < 0.05, ***p* < 0.01. (F) The cell viability of H_2_O_2_-induced HCECs on the groups of PBS, TL, INS, INS-lip, TI, L, and siVEGF-TIL was also verified by CCK-8 assay. Data are presented as mean ± SD (n = 3 per group). **p* < 0.05. TL: Trimethyl Chitosan coated liposome; siVEGF-TL: siVEGF-trimethyl chitosan coated liposome; INS: insulin; INS-lip: insulin liposome; TIL: trimethyl chitosan coated insulin liposome; siVEGF-TIL: siVEGF-trimethyl chitosan coated insulin liposome.


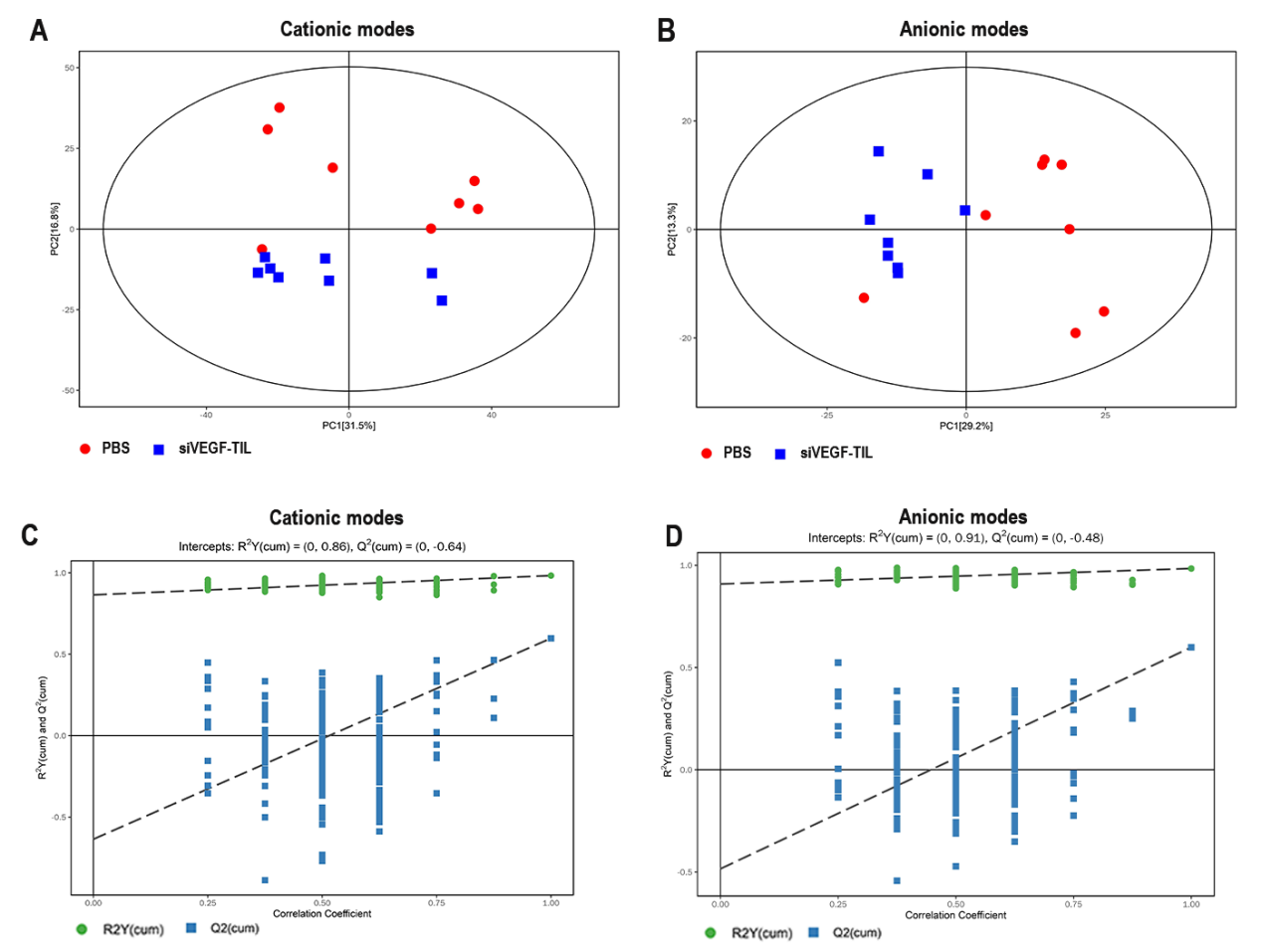


**Figure S4.** PCA analysis of the included samples in PBS and INS groups under the cationic (A) and anionic mode (B). Permutation analysis plot of the OPLS-DA model, under the cationic (C) and anionic mode (D). PCA: principal component analysis; OPLS-DA: orthogonal projection to latent structure-discriminant analysis; INS: insulin.

**Table S2.** Significantly Altered Metabolites by Untargeted Metabolomics Under anionic mode

| Metabolite | VIP | Fold Change | *P* Value | Mean A | Mean B |
| --- | --- | --- | --- | --- | --- |
| Glycolic acid | 1.63 | 1.60 | 0.00 | 0.09 | 0.06 |
| L-Norleucine | 1.54 | 1.65 | 0.01 | 0.36 | 0.22 |
| Linoelaidic acid | 1.86 | 1.66 | 0.00 | 5.86 | 3.54 |
| Eicosadienoic acid | 1.14 | 2.15 | 0.00 | 0.47 | 0.22 |
| Adrenic acid | 1.26 | 1.37 | 0.04 | 1.20 | 0.87 |
| L-Alanine | 1.08 | 1.35 | 0.04 | 1.04 | 0.77 |
| Heptadecanoic acid | 1.44 | 1.36 | 0.03 | 0.47 | 0.34 |
| Gamma-Linolenic acidγ | 1.21 | 1.47 | 0.04 | 0.13 | 0.09 |
| L-Proline | 1.40 | 1.56 | 0.03 | 0.19 | 0.12 |
| Xanthine | 1.68 | 1.80 | 0.01 | 0.43 | 0.24 |
| 16-Hydroxy hexadecanoic acid | 1.56 | 1.67 | 0.01 | 0.20 | 0.12 |
| Cytidine | 1.41 | 1.57 | 0.04 | 0.17 | 0.11 |
| 2-Oxovaleric acid | 1.65 | 1.31 | 0.01 | 0.93 | 0.83 |
| L-Valine | 1.43 | 1.69 | 0.04 | 0.16 | 0.10 |
| D-Xylose | 1.60 | 1.57 | 0.01 | 0.29 | 0.18 |
| N-Acetyl-L-alanine | 1.33 | 2.82 | 0.01 | 0.01 | 0.00 |
| (10E,12Z)-(9S)-9-Hydroperoxyoctadeca-10,12-dienoic acid | 1.61 | 2.94 | 0.02 | 0.10 | 0.03 |
| Phenylpyruvic acid | 1.54 | 1.17 | 0.01 | 0.26 | 0.22 |
| Pyroglutamic acid | 1.45 | 1.26 | 0.02 | 0.03 | 0.03 |
| 3-Phosphoglyceric acid | 1.75 | 2.97 | 0.00 | 0.03 | 0.01 |
| L-Phenylalanine | 1.30 | 1.57 | 0.04 | 0.22 | 0.14 |
| Allantoin | 1.40 | 1.33 | 0.02 | 2.02 | 1.52 |
| 2-Pyrocatechuic acid | 1.32 | 2.03 | 0.01 | 0.02 | 0.01 |
| D-Glutamine | 1.08 | 1.78 | 0.04 | 0.04 | 0.02 |
| Dimethylmalonic acid | 1.26 | 1.12 | 0.05 | 0.03 | 0.02 |
| N-Acetyl-L-methionine | 1.90 | 2.25 | 0.01 | 0.08 | 0.03 |
| Xanthosine | 1.89 | 2.63 | 0.01 | 0.05 | 0.02 |
| LysoPA(16:0/0:0) | 1.95 | 1.74 | 0.00 | 0.00 | 0.00 |
| L-Glutamic acid | 1.21 | 1.33 | 0.04 | 0.04 | 0.03 |
| N-Acetylgalactosamine 6-sulfate | 1.85 | 2.05 | 0.01 | 2.23 | 1.09 |
| L-Methionine | 1.44 | 2.80 | 0.02 | 0.08 | 0.03 |
| 2-Oxo-4-methylthiobutanoic acid | 2.00 | 5.30 | 0.00 | 0.11 | 0.02 |
| Adenine | 1.90 | 0.50 | 0.00 | 0.06 | 0.12 |
| gamma-Aminobutyric acid | 1.83 | 0.39 | 0.00 | 0.02 | 0.05 |
| Adenosine | 1.56 | 0.48 | 0.01 | 0.23 | 0.48 |
| Malonic acid | 1.73 | 0.79 | 0.00 | 0.06 | 0.07 |
| L-Iditol | 1.97 | 0.51 | 0.00 | 0.04 | 0.08 |
| D-Xylose | 1.67 | 0.51 | 0.00 | 0.29 | 0.18 |
| Uracil | 1.99 | 0.74 | 0.00 | 0.10 | 0.03 |
| Pantothenol | 1.41 | 0.26 | 0.03 | 0.01 | 0.04 |
| Orotidine | 1.86 | 0.53 | 0.00 | 0.04 | 0.08 |
| 2-Hydroxyethanesulfonate | 2.24 | 0.51 | 0.00 | 0.41 | 0.81 |
| Thioguanine | 1.74 | 0.45 | 0.00 | 0.31 | 0.69 |
| Sucrose | 1.50 | 0.57 | 0.02 | 0.04 | 0.06 |
| Azelaic acid | 1.40 | 0.72 | 0.02 | 0.02 | 0.02 |
| Fexofenadine | 1.42 | 0.61 | 0.01 | 0.01 | 0.02 |
| p-Toluenesulfonic acid | 1.51 | 0.45 | 0.04 | 0.01 | 0.02 |
| 3-Hydroxymethylglutaric acid | 1.25 | 0.58 | 0.01 | 0.02 | 0.04 |

Compared with the INS group, red stands for up-regulated metabolomics and blue stands for down-regulated in the PBS group.

**Table S3.** Significantly Altered KEGG Pathway by Untargeted Metabolomics Under Anionic Mode

| **KEGG Pathway** | **Compounds** | **Group** | **DA** |
| --- | --- | --- | --- |
| Carbon metabolism | L-Alanine, L-Glutamate | unknown | 1 |
| 2-Oxocarboxylic acid metabolism | L-Valine, L-Phenylalanine, L-Glutamate, L-Methionine,  4-Methylthio-2-oxobutanoic acid | unknown | 1 |
| Biosynthesis of amino acids | Alanine, L-Proline, L-Valine, L-Phenylalanine, L-Glutamate, L-Methionine | unknown | 1 |
| Metabolic pathways | Adenine, L-Alanine, Gamolenic acid, L-Proline, Xanthine, Cytidine, Adenosine,Malonate, L-Valine, D-Xylose, Uracil, L-Phenylalanine, Orotidylic acid, D-Glutamine, Sucrose, Xanthosine, Phosphatidate, L-Glutamate, L-Methionine, 4-Methylthio-2-oxobutanoic acid | unknown | 0.4 |
| Aminoacyl-tRNA biosynthesis | L-Alanine, L-Proline, L-Valine, L-Phenylalanine, L-Glutamate, L-Methionine | Translation | 1 |
| Alcoholism | Adenosine, L-Glutamate | Substance dependence | 0 |
| Neuroactive ligand-receptor interaction | Adenosine, L-Glutamate | Signaling molecules and interaction | 0 |
| cAMP signaling pathway | Adenosine, Phosphatidate | Signal transduction | 0 |
| Phospholipase D signaling pathway | Phosphatidate, L-Glutamate | Signal transduction | 1 |
| Taste transduction | Sucrose, L-Glutamate | Sensory system | 0 |
| Purine metabolism | Adenine, Xanthine, Adenosine, Xanthosine | Nucleotide metabolism | 0 |
| Pyrimidine metabolism | Cytidine, Malonate, Uracil, Orotidylic acid | Nucleotide metabolism | -0.5 |
| beta-Alanine metabolism | Malonate, Uracil | Metabolism of other amino acids | -1 |
| Taurine and hypotaurine metabolism | L-Alanine, L-Glutamate | Metabolism of other amino acids | 1 |
| D-Glutamine and D-glutamate metabolism | D-Glutamine, L-Glutamate | Metabolism of other amino acids | 1 |
| Pantothenate and CoA biosynthesis | L-Valine, Uracil, Pantothenol | Metabolism of cofactors and vitamins | -0.33 |
| ABC transporters | L-Alain, L-Proline, Cytidine, Adenosine, L-Valine, D-Xylose, L-Phenylalanine, Sucrose, Xanthosine, L-Glutamate | Membrane transport | 0.6 |
| Biosynthesis of unsaturated fatty acids | Icosadienoic acid, Adrenic acid, gamma-Linolenic acid | Lipid metabolism | 1 |
| Mineral absorption | L-Alanine, L-Proline, L-Valine, L-Phenylalanine, L-Methionine | Digestive system | 1 |
| Protein digestion and absorption | L-Alanine, L-Proline, L-Valine, L-Phenylalanine, L-Glutamate, L-Methionine | Digestive system | 1 |
| Ferroptosis | Adrenic acid, L-Glutamate | Cell growth and death | 1 |
| Central carbon metabolism in cancer | L-Alanine, L-Proline, L-Valine, L-Phenylalanine, L-Glutamate, L-Methionine | Cancer: Overview | 1 |
| Caffeine metabolism | Xanthine, Xanthosine | Biosynthesis of other secondary metabolites | 1 |
| Alanine, aspartate and glutamate metabolism | L-Alanine, L-Valine | Amino acid metabolism | 1 |
| Arginine and proline metabolism | L-Proline, L-Valine | Amino acid metabolism | 1 |
| Cysteine and methionine metabolism | L-Alanine, L-Methionine, 4-Methylthio-2-oxobutanoic acid | Amino acid metabolism | 1 |

DA: differential abundance score

**Table S4** Primers sequences

| **Gene** | **Primer sequence (5’→3′)** |
| --- | --- |
| VEGF | Forward GGCCTCCGAAACCATGAACT |
|  | Reverse GCAGTAGCTGCGCTGATAGA |
